# Bifidobacterial Dominance of the Gut in Early Life and Acquisition of Antimicrobial Resistance

**DOI:** 10.1128/mSphere.00441-18

**Published:** 2018-09-26

**Authors:** Diana H. Taft, Jinxin Liu, Maria X. Maldonado-Gomez, Samir Akre, M. Nazmul Huda, S. M. Ahmad, Charles B. Stephensen, David A. Mills

**Affiliations:** a Department of Food Science and Technology, University of California, Davis, Davis, California, USA; b Foods for Health Institute, University of California, Davis, Davis, California, USA; c Nutrition Department, University of California, Davis, Davis, California, USA; d Western Human Nutrition Research Center, U.S. Department of Agriculture, Davis, California, USA; e Enteric and Respiratory Infections Unit, Infectious Diseases Division, ICDDR,B, Dhaka, Bangladesh; f Department of Viticulture and Enology, University of California, Davis, Davis, California, USA; University of Wisconsin—Madison

**Keywords:** antimicrobial resistance, bifidobacteria, gut metagenome, infant

## Abstract

*Bifidobacterium* species are important commensals capable of dominating the infant gut microbiome, in part by producing acids that suppress growth of other taxa. *Bifidobacterium* species are less prone to possessing antimicrobial resistance (AMR) genes (ARGs) than other taxa that may colonize infants. Given that AMR is a growing public health crisis and ARGs are present in the gut microbiome of humans from early life, this study examines the correlation between a *Bifidobacterium-*dominated infant gut microbiome and AMR levels, measured by a culture-independent metagenomic approach both in early life and as infants become toddlers. In general, *Bifidobacterium* dominance is associated with a significant reduction in AMR in a Bangladeshi cohort, both in the number of acquired AMR genes present and in the abundance of AMR genes. However, by year 2, Bangladeshi infants had no significant differences in AMR related to their early-life *Bifidobacterium* levels. A generalized linear model including all infants in a previously published Swedish cohort found a significant negative association between log-transformed total AMR and *Bifidobacterium* levels, thus confirming the relationship between *Bifidobacterium* levels and AMR. In both cohorts, there was no change between early-life and later-life AMR abundance in high-*Bifidobacterium* infants but a significant reduction in AMR abundance in low-*Bifidobacterium* infants. These results support the hypothesis that early *Bifidobacterium* dominance of the infant gut microbiome may help reduce colonization by taxa containing ARGs.

**IMPORTANCE** Infants are vulnerable to an array of infectious diseases, and as the gut microbiome may serve as a reservoir of AMR for pathogens, reducing the levels of AMR in infants is important to infant health. This study demonstrates that high levels of *Bifidobacterium* are associated with reduced levels of AMR in early life and suggests that probiotic interventions to increase infant *Bifidobacterium* levels have the potential to reduce AMR in infants. However, this effect is not sustained at year 2 of age in Bangladeshi infants, underscoring the need for more detailed studies of the biogeography and timing of infant AMR acquisition.

## Abstract (precis)

Infants are vulnerable to an array of infectious diseases, and as the gut microbiome may serve as a reservoir of AMR for pathogens, reducing the levels of AMR in infants is important to infant health. This study demonstrates that high levels of *Bifidobacterium* are associated with reduced levels of AMR in early life and suggests that probiotic interventions to increase infant *Bifidobacterium* levels have the potential to reduce AMR in infants. However, this effect is not sustained at year 2 of age in Bangladeshi infants, underscoring the need for more detailed studies of the biogeography and timing of infant AMR acquisition.

## INTRODUCTION

*Bifidobacterium* species are key members of the infant gut microbiome, and some species are capable of completely dominating the breastfed infant gut (1). This dominance of the infant gut by *Bifidobacterium* is linked to consumption of human milk oligosaccharide (HMO); some species of *Bifidobacterium*, notably Bifidobacterium longum subsp. *infantis*, consume HMO more efficiently than others (2–5). *Bifidobacterium* ferments HMOs into, among other products, lactate and acetate (6), which reduces luminal pH and inhibits the growth of pathogens and other bacterial taxa in the gut (4, 5, 7). However, the proportion of infants with *Bifidobacterium-*dominated gut microbiomes appears to vary drastically between countries (8), with developing countries such as Bangladesh (9), Malawi (10), Gambia (11), and Kenya (12) having a higher proportion of infants with higher *Bifidobacterium* levels than more developed countries such as Finland (10), Sweden (13), and the United States (14). A recent clinical trial found that providing breastfed infants in the United States with an HMO-consuming *Bifidobacterium* probiotic resulted in lasting colonization of the infant, with levels of *Bifidobacterium* closer to those seen in developing countries than in the control group (4). With the introduction of complementary feeding, natural levels of *Bifidobacterium* in the infant gut decrease (15), but the influence of early-life *Bifidobacterium* dominance on health extends past the period in early life to provide lasting benefit by improving vaccine response (9), reducing risk of obesity, (16) and reducing risk of allergy (17).

This dominance of the infant gut with *Bifidobacterium* during early life may have important implications for infant antimicrobial resistance (AMR) acquisition because of the relatively low occurrence of AMR in *Bifidobacterium* (18, 19). While *Bifidobacterium* is intrinsically resistant to several antibiotics, it is not known to carry transferable antimicrobial resistance genes (ARGs) with the notable exception of *tet*(W) (18, 20). Furthermore, there is some evidence that *Bifidobacterium* may reduce the transfer of beta-lactamase resistance genes between different species of *Enterobacteriaceae* (21). Therefore, a *Bifidobacterium-*dominated infant gut can restrict the growth of other taxa, and the susceptibility of *Bifidobacterium* to antimicrobials creates the possibility that a *Bifidobacterium-*dominated gut may also reduce levels of ARGs during the assembly of the gut microbiome community as *Bifidobacterium* outcompetes species more prone to AMR. This potential reduction of AMR is relevant because AMR is a substantial and growing public health crisis both in the United States and globally. AMR can arise from a variety of human activities, including therapeutic use of antibiotics in human and animals and growth promotion of food-producing animals (22). The increased AMR in the biosphere results in increased probability of transmission to humans. As AMR becomes more common, even infants are beginning to harbor antimicrobial resistance genes (ARGs)—ARGs have been detected in infant meconium (23). The carriage of AMR organisms carries risks for human health; harboring AMR organisms correlates with an increased risk of severe infection and death (24–26). This is evident at the population level as well as the individual level. At this juncture, the AMR crisis has reached levels such that, in the United States, AMR contributes to more than 2,000,000 illnesses and a minimum of 23,000 deaths each year (27).

The timing of AMR acquisition by neonates and AMR persistence throughout the first 1,000 days of life are a key health care concern. A prior study of AMR in infants not exposed to antimicrobial agents over the 1st year of life found that erythromycin, sulfonamide, and cefotaxime resistance were found by day 1 of life and tetracycline resistance was detected by day 3 of life. However, the ARGs found in infants did not always match those found in mothers (28). This same study also reported a rapid increase in ARGs during the first few months of life (28). A twin study reported that the infant resistome was different from the maternal resistome by 2 months of age and also reported that resistance proteins were more likely to be found at a single time point in an individual infant than to persist at multiple time points, suggesting that the environment is an important source of ARGs in infants (29). Here, we examined if having high levels of *Bifidobacterium* in early infancy correlated with reduced levels of AMR in the infant gut microbiome and explored if such correlations extended into the weaning period. To do this, this study examined the association of *Bifidobacterium* levels in early life with the acquisition of AMR both when an infant may be *Bifidobacterium* dominated and later in life using a cohort from a developing country (Bangladesh) with a high prevalence of *Bifidobacterium* dominance of the infant gut microbiome (9) and a cohort from a more developed country (Sweden) where *Bifidobacterium* dominance of the infant gut microbiome is less common (30).

## RESULTS

### Bangladeshi infants.

**FIG 1:**
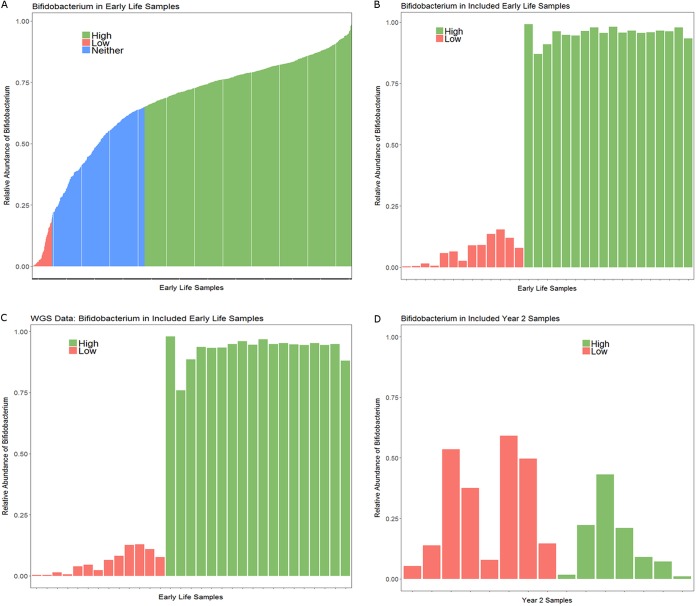
Relative abundance of *Bifidobacterium* in early-life Bangladeshi samples. (A) Relative abundance of *Bifidobacterium* in all early-life samples from Bangladesh by 16S rRNA gene sequencing, up to three samples per infant included. Of 849 samples, 550 samples were high *Bifidobacterium* and 53 samples were low *Bifidobacterium*. (B) Relative abundance of *Bifidobacterium* in early-life samples included in AMR analysis by 16S rRNA gene sequencing, only one sample per infant included. (C) Relative abundance of *Bifidobacterium* in early-life samples included in AMR analysis using WGS data as analyzed by METAXA2; samples are in the same order as in panel B. (D) Relative abundance of *Bifidobacterium* at year 2. No year 2 sample had high-enough *Bifidobacterium* levels to be classified as high *Bifidobacterium*.

Infant samples were selected for whole-metagenomic sequencing (WGS) from a prior study of vitamin A supplementation and vaccine efficacy based on the level of *Bifidobacterium* present during early life (9). As part of that study, infant stool samples were sequenced in early life (6, 11, and 15 weeks) and year 2 using 16S rRNA sequencing. This original study was then expanded to include 306 infants, 291 of whom provided at least one stool sample in early life and 249 of whom had a year 2 stool sample sequenced (31). Of the 291 infants with at least one early-life stool sample, 60% were delivered by Caesarean section. The relative abundance of *Bifidobacterium* in each sample was calculated using the 16S rRNA sequencing data, and early-life samples were chosen as follows: all available low-*Bifidobacterium* samples (<20% *Bifidobacterium* relative abundance; threshold selected because the distribution of *Bifidobacterium* in the cohort was bimodal, with a major peak around 80% and a second peak under 20%) with sufficient DNA for sequencing were included. High-*Bifidobacterium* samples (>65% relative abundance; threshold selected because 65% was the mean abundance of *Bifidobacterium*) were chosen by selecting the highest-*Bifidobacterium* sample with sufficient DNA remaining for sequencing which would maintain approximate gender balance with the low-*Bifidobacterium* samples (Fig. 1A and B). To assess the stability of the *Bifidobacterium* groups, the *Bifidobacterium* levels at week 6, 11, and 15 samples were compared in individual infants. The study (31) included 287 infants with multiple early-life samples. Of these infants, 42% (121 infants) of infants had a stable colonization pattern: 35% (101 infants) had samples included in the high-*Bifidobacterium* group at all sequenced time points, 6% (17 infants) had samples that consistently fell in the intermediate-*Bifidobacterium* group, and 1% (3 infants) had samples that were classified as low *Bifidobacterium* at all time points. In addition, 22% of infants (63 infants) transitioned from a lower level of *Bifidobacterium* to a higher level: 17% (50 infants) transitioned from the intermediate group at the earliest time point to the high group, 2% (5 infants) transitioned from the low-*Bifidobacterium* group to the intermediate group, and 3% (8 infants) transitioned from the low group at the earliest time point to the high group at the last time point. Seventeen percent of infants (48 infants) showed decreased *Bifidobacterium* over time: 15% (42 infants) transitioned from the high group to the intermediate group, 1% (2 infants) transitioned from the high group to the low group, and 1% (4 infants) transitioned from the intermediate group to the low group. Sixteen percent of infants (45 infants) fluctuated between two categories: 13% (37 infants) fluctuated between the high and intermediate groups, 2% (5 infants) fluctuated between the low and intermediate groups, and 1% (3 infants) fluctuated between the low and high groups. The remaining 3% of infants had 1 sample in each of the three categories. Volatility is a normal feature of the infant gut microbiome (32), and as such, some fluctuation between *Bifidobacterium* groups over time is anticipated. The greater stability of infants with high levels of *Bifidobacterium* is consistent with the literature, which shows that infants given a Bifidobacterium longum subsp. *infantis* probiotic in the 1st week of life tend to have greater stability of the microbiome than infants who received the placebo (5). Furthermore, the high levels of *Bifidobacterium* circulating in this population likely provide ample opportunity for infants to acquire *Bifidobacterium* at later ages, perhaps explaining why 22% of infants transitioned to higher levels of *Bifidobacterium* at a later time point. To avoid repeated-measure issues in the whole-metagenomic sequencing, only one early-life sample per infant was eligible for inclusion. For infants with multiple low-*Bifidobacterium* samples available, a sample was chosen at random for inclusion. For high-*Bifidobacterium* samples, the sample with the highest relative abundance of *Bifidobacterium* that would maintain close to gender parity with the low-*Bifidobacterium* samples was selected. The high prevalence of *Bifidobacterium* colonization in the Bangladeshi infants limited the number of infants with samples that met the criteria of low *Bifidobacterium* (<20% relative abundance of *Bifidobacterium*). As a result, all samples that met the criteria for low *Bifidobacterium* and had sufficient DNA for WGS were included (a total of 13 samples). These low-*Bifidobacterium* samples included one sample from each of the 3 infants who exhibited stable low-*Bifidobacterium* relative abundance, 4 samples from infants who initially had low *Bifidobacterium* levels and later had high *Bifidobacterium* levels, 2 samples from infants who transitioned from the low-*Bifidobacterium* group to the intermediate group, 3 samples from infants who fluctuated between the low and intermediate groups, and a single sample from an infant who had samples in each of the three categories. For high *Bifidobacterium*, 18 early-life high-*Bifidobacterium* (>65% relative abundance of *Bifidobacterium*) samples were sequenced. The majority of the included high-*Bifidobacterium* samples (13 samples) were from infants with a stable high-colonization pattern, with 3 samples coming from infants who fluctuated between the high-*Bifidobacterium* and intermediate categories and 2 samples coming from infants who transitioned from the high group to the intermediate group at later time points. This resulted in a total of 31 early-life infant samples for inclusion. Of these 31 infants, 15 infants had a year 2 sample with sufficient DNA available for WGS. High- and low-*Bifidobacterium* infants were similar in gender and week of early-life sample collection (Table 1). METAXA2 v. 2.1.3 was used to confirm the relative abundance of *Bifidobacterium* in the early-life samples (Fig. 1C). As the lowest number of reads mapping to the large and small rRNA subunit from a sample was 18,597, the METAXA2 result read depth was rarefied to 18,597 reads for all samples prior to estimating alpha diversity using both the Shannon index and the Chao1 richness index. In early life, high-*Bifidobacterium* samples had significantly lower alpha diversity by both the Shannon index (Kruskal-Wallis test, *P* < 0.0001) and the Chao1 richness index (Kruskal-Wallis test, *P* = 0.0002). In the later-life samples, there was no significant difference in alpha diversity between infants who were high or low *Bifidobacterium* in early life by either the Shannon index (Kruskal-Wallis test, *P* = 0.16) or the Chao1 richness index (Kruskal-Wallis test, *P* = 0.30). However, the later-life samples did have a significantly greater alpha diversity by both the Shannon index (Wilcoxon paired test, *P* = 0.003) and the Chao1 richness index (Wilcoxon paired test, *P* < 0.0001) (see Fig. S1 in the supplemental material). There were significant differences in delivery mode between high- and low-*Bifidobacterium* infants (chi-square test, *P* = 0.00956), with 15 of the 18 high-*Bifidobacterium* infants delivered by Caesarean section compared to 4 of 13 low-*Bifidobacterium* infants delivered by Caesarean section.

**TABLE 1:** Characteristics of Bangladeshi infants with high- and low-*Bifidobacterium* samples in early life^*a*^

*Bifidobacterium* level in early life	No. of samples from:		No. of male infants		No. of C-section deliveries		Early-life sample wk of collection, wk 6/wk 11/wk 15	Mean wk of early-life sample collection
	Early life	Yr 2	Early life	Yr 2	Early life	Yr 2		
High	18	7	12	3	15	5	8/7/3	9.4
Low	13	8	8	4	4	1	8/4/1	8.2
*P* value			*P* = 1	*P* = 1	*P* = 0.0096	*P* = 0.072	*P* = 0.59	

a *P* values were calculated using a chi-square test; a *P* value of 0.05 was used for significance.

10.1128/mSphere.00441-18.1 FIG S1 Alpha diversity measured by the Shannon index and the Chao1 richness index calculated using METAXA2 results. (A) Early-life Shannon index. (B) Early-life Chao1 richness index. (C) Later-life Shannon index. (D) Later-life Chao1 richness index. (E) Early versus late Shannon index. (F) Early versus late Chao1 richness index. As two different metrics were used for alpha diversity, a *P* value of 0.025 was used for significance. Download FIG S1, PDF file, 0.3 MB. Copyright © 2018 Taft et al. 2018 Taft et al. This content is distributed under the terms of the Creative Commons Attribution 4.0 International license.

### Metagenomic sequencing results.

**FIG 2:**
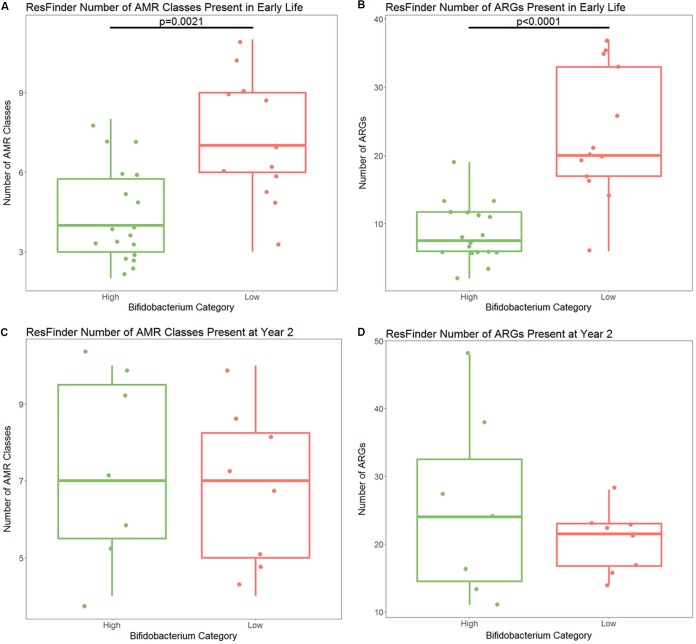
(A) Number of AMR classes with at least one ARG detected by ResFinder in high- and low-*Bifidobacterium* samples in early life. There are significantly fewer AMR classes present in high-*Bifidobacterium* samples (*P* = 0.0021). (B) Number of different ARGs detected by ResFinder in early life. There are significantly fewer ARGs present in high-*Bifidobacterium* samples (*P* < 0.0001). (C) Number of different AMR classes at year 2; there is no significant difference by *Bifidobacterium* levels in early life (*P* = 0.725). (D) Number of different ARGs detected by ResFinder at year 2. There is no significant difference by *Bifidobacterium* levels in early life (*P* = 0.685). All statistical testing was performed using a Kruskal-Wallis test. *P* value for significance is 0.025.

The Bangladeshi samples were sequenced in three separate lanes in three separate runs. Runs 1 and 2 contained a mixture of high- and low-*Bifidobacterium* samples from early life. Run 3 contained all year 2 samples. There were no significant differences in merged read depth by run (analysis of variance [ANOVA], *P* = 0.92) (Table S1). There were no significant differences in the number of contigs generated from samples in run 1 and run 2 (Kruskal-Wallis test, *P* = 0.87); however, there were significantly more contigs generated for low-*Bifidobacterium* early-life samples than for high-*Bifidobacterium* early-life samples. Run 3 did have significantly more contigs than runs 1 and 2 (Kruskal-Wallis test comparing runs 1 and 3 and comparing runs 2 and 3, both *P* < 0.0001), likely related to the increased diversity of the microbiome between the early-life and later-life samples. Table S1 presents the read depth, number of contigs produced, the total contig length, and the N50 for each sample. After merging paired-end reads, sequencing depth was significantly greater in high-*Bifidobacterium* early-life samples than in low-*Bifidobacterium* early-life samples (*t* test, *P* = 0.002, mean 16,156,413 reads in high-*Bifidobacterium* samples and mean 13,672,554 reads in low-*Bifidobacterium* samples) (Fig. S2). However, the number of contigs and the total assembly length were significantly lower in high-*Bifidobacterium* early-life samples than in low-*Bifidobacterium* early-life samples (Kruskal-Wallis test; contig count, *P* < 0.0001; total length, *P* < 0.0001). This is consistent with the alpha diversity analysis which found lower diversity in the high-*Bifidobacterium* samples, as a less diverse microbiome will have fewer different genomes present and will therefore have a shorter total assembly length. If bias exists, the lower number of reads in the low-*Bifidobacterium* samples will tend to bias results toward finding an increased number of ARGs in high-*Bifidobacterium* samples. Comparison to the ResFinder database with default settings revealed that high-*Bifidobacterium* samples in early life had significantly fewer classes of ARGs present than low-*Bifidobacterium* samples (*P* = 0.0021) (Fig. 2A). High-*Bifidobacterium* samples had a median of 4 ARG classes present per sample (range of 2 to 8 ARG classes per sample) compared to low-*Bifidobacterium* samples, which had a median of 7 ARG classes present per sample (range of 3 to 11 ARG classes per sample).

10.1128/mSphere.00441-18.2 FIG S2 High-*Bifidobacterium* samples in early life had a significantly higher merged read depth than low-*Bifidobacterium* samples in early life. This will tend to bias the results toward detecting more ARGs in high-*Bifidobacterium* samples in the early-life analysis. A *P* value of 0.05 was used for significance. Download FIG S2, PDF file, 0.1 MB. Copyright © 2018 Taft et al. 2018 Taft et al. This content is distributed under the terms of the Creative Commons Attribution 4.0 International license.

10.1128/mSphere.00441-18.6 TABLE S1 Bangladeshi sequencing results. Download Table S1, PDF file, 0.1 MB. Copyright © 2018 Taft et al. 2018 Taft et al. This content is distributed under the terms of the Creative Commons Attribution 4.0 International license.

Looking across all early-life samples using ResFinder, there are a total of 11 classes of ARGs present: aminoglycoside; beta-lactam; fluoroquinolone; fosfomycin; macrolide, lincosamide, and streptogramin B (MLS); phenicol; sulfonamide; tetracycline; trimethoprim; glycopeptide; and nitroimidazole. All 11 of these classes were present in the low-*Bifidobacterium* samples, but only 9 of the classes were present in high-*Bifidobacterium* samples. The classes present in the low-*Bifidobacterium* early-life samples but absent in high-*Bifidobacterium* early-life samples were glycopeptide resistance (present in 2 low-*Bifidobacterium* samples) and nitroimidazole (present in 1 low-*Bifidobacterium* sample). All other classes were detected in at least 9 of the 31 samples. Only a single class, MLS, was present in all samples (Table S2).

10.1128/mSphere.00441-18.7 TABLE S2 Presence of transferable AMR classes in high- and low-*Bifidobacterium* samples. Download Table S2, PDF file, 0.1 MB. Copyright © 2018 Taft et al. 2018 Taft et al. This content is distributed under the terms of the Creative Commons Attribution 4.0 International license.

High-*Bifidobacterium* samples in early life also had significantly fewer transferable ARGs present (*P* < 0.0001) (Fig. 2B). This difference became greater after dividing the number of ARGs present per sample by the number of merged reads included in the assembly per sample, supporting the idea that the difference is not due to differences in sequencing depth (*P* < 0.0001) (Fig. S3). High-*Bifidobacterium* samples had a median of 7.5 ARGs per sample (range, 2 to 19), and low-*Bifidobacterium* samples had a median of 20 ARGs per sample (range, 6 to 37). There were a total of 108 ARGs detected by ResFinder in early-life samples. Of these, 103 were present in low-*Bifidobacterium* samples and 50 were present in high-*Bifidobacterium* samples (Table 2). Of the 50 ARGs present only in the high-*Bifidobacterium* samples, five were present in only a single high-*Bifidobacterium* sample. These ARGs were *str* (aminoglycoside resistance), *aadA1* (aminoglycoside resistance), *cat*(pc221) (phenicol resistance), *bla*_LEN12_ (beta-lactam resistance), and *strA* (aminoglycoside resistance). These genes were found in 4 separate samples, with *aadA1* and *strA* detected in the same sample. The five genes found only in high-*Bifidobacterium* samples stand in contrast to 58 ARGs found only in low-*Bifidobacterium* samples. Of these 58 genes, 41 were found in only a single low-*Bifidobacterium* sample, and 11 were found in two low-*Bifidobacterium* samples. Due to our small cohort size, an ARG present in only one low-*Bifidobacterium* sample would have a prevalence of 8% in low-*Bifidobacterium* infants and 3% in all infants. There was a median of 2 unique ARGs per low-*Bifidobacterium* sample (range, 0 to 7 ARGs per sample). Two genes were found in three low-*Bifidobacterium* samples: one was a beta-lactam resistance gene, *bla*_SHV-1_, and the other was a tetracycline resistance gene, *tet*(31). Two genes were found only in 4 of the 15 low-*Bifidobacterium* samples: one was a beta-lactam resistance gene, *bla*_ACI-1_, and one was a tetracycline resistance gene, *tet*(Q). Two genes were found only in 5 of the 15 low-*Bifidobacterium* samples, an MLS resistance gene, *erm*(T), and a fluoroquinolone resistance gene, *oqxB*. There was not a significant difference between ARG count and delivery mode in early life in either the high-*Bifidobacterium* infants (Kruskal-Wallis test, *P* = 0.40) or the low-*Bifidobacterium* infants (Kruskal-Wallis test, *P* = 0.76).

10.1128/mSphere.00441-18.3 FIG S3 Differences in number of ARGs per sample divided by read depth per sample in high- and low-*Bifidobacterium* samples. High-*Bifidobacterium* samples still had significantly fewer ARGs than low-*Bifidobacterium* samples (*P* < 0.0001). A *P* value of 0.025 was used for significance. Download FIG S3, PDF file, 0.1 MB. Copyright © 2018 Taft et al. 2018 Taft et al. This content is distributed under the terms of the Creative Commons Attribution 4.0 International license.

**TABLE 2:** Number of ARGs present in Bangladeshi samples by ResFinder

*Bifidobacterium* level	Time	No. of ARGs			
		Total present	Unique to *Bifidobacterium* level	Detected in only a single sample	Median per sample (range)
High	Early life	50	5	5	7.5 (2–19)
Low	Early life	103	58	41	20 (6–37)
High	Yr 2	73	27	23	24 (11–48)
Low	Yr 2	62	16	12	21.5 (14–28)

NCBI BLAST (33), in combination with a custom python script (https://github.com/akre96/ResistBlast), was used to attempt to predict the taxa of origin of contigs identified as containing an ARG by ResFinder. The family *Enterobacteriaceae* was the most common predicted origin of ARGs in both high- and low-*Bifidobacterium* samples, accounting for an average of 49% of the ARGs per sample detected in the high-*Bifidobacterium* samples and 43% of the ARGs per sample detected in the low-*Bifidobacterium* samples. In the high-*Bifidobacterium* samples, other detected families of origin in decreasing average percentage of ARGs per sample were as follows: *Enterococcaceae* (34% of ARGs on average), *Staphylococcaceae* (30% of ARGs on average), *Streptococcaceae* (29% of ARGs on average), *Bifidobacteriaceae* (19% of ARGs on average), mixed (meaning that the top three hits from BLAST results belonged to multiple families; 19% of ARGs on average), unknown (meaning either no hits by BLAST or that all hits corresponded to uncultured bacteria; 17% of ARGs on average), and *Actinomycetaceae* (7.7% of ARGs on average). All families detected in the high-*Bifidobacterium* samples were also detected in the low-*Bifidobacterium* samples, but the low-*Bifidobacterium* samples identified ARGs originating in additional taxa. In the low-*Bifidobacterium* samples, detected families of origin, in decreasing average percentage of ARGs per sample, were as follows: mixed (20% of ARGs on average), *Campylobacteraceae* (19% of ARGs on average), *Staphylococcaceae* (12% of ARGs on average), *Enterococcaceae* (11% of ARGs on average), unknown (11% of ARGs on average), *Moraxellaceae* (10% of ARGs on average), *Streptococcaceae* (10% of ARGs on average), *Bifidobacteriaceae* (6.0% of ARGs on average), *Peptostreptococcaceae* (5.9% of ARGs on average), *Bacteroidaceae* (5.8% of ARGs on average), *Flavobacteriaceae* (5.0% of ARGs on average), *Prevotellaceae* (2.8% of ARGs on average), and *Actinomycetaceae* (2.7% of ARGs on average).

There were no significant differences in merged read depth of the later-life (year 2) samples between infants who were high *Bifidobacterium* in early life and those who were low *Bifidobacterium* in early life (*t* test, *P* = 0.90). By year 2, all infants included had <65% relative abundance of *Bifidobacterium* (Fig. 1D), an expected result as *Bifidobacterium* levels are known to decrease with the introduction of complementary foods and to fall further when children are completely weaned (15). The relatively high *Bifidobacterium* levels (>50%) in some infants may reflect the fact that by 2 years of age, 80% of Bangladeshi women are still providing some breast milk to their child (34). At year 2, ResFinder detected no significant difference in the number of AMR classes or the number of ARGs detected in samples from infants who had high levels of *Bifidobacterium* in early life compared to infants who had low levels of *Bifidobacterium* in early life (Kruskal-Wallis test, *P* = 0.725 for AMR class and *P* = 0.685 for ARGs) (Fig. 2C and D). The same 11 classes of transferable ARGs present in low-*Bifidobacterium* samples in early life were detected at year 2 in both high- and low-*Bifidobacterium* samples: aminoglycoside, beta-lactam, fluoroquinolone, fosfomycin, glycopeptide, MLS, nitroimidazole, phenicol, sulfonamide, tetracycline, and trimethoprim. At year 2, 89 ARGs were detected in at least one sample. There were 73 different ARGs detected in samples from infants with high *Bifidobacterium* in early life but only 62 different ARGs detected in samples from infants with low *Bifidobacterium* (Table 2). Of the 27 ARGs found only in samples from infants who had high *Bifidobacterium* in early life, 23 were found in only one sample, 3 were found in two samples, and one gene (*cfxA4*, beta-lactam resistance) was present in 3 samples. Of the 16 ARGs found only in samples from infants who had low *Bifidobacterium* in early life, 12 were found in only one sample and 4 were found in two samples.

At year 2, *Enterobacteriaceae* remained the most common predicted origin of ARGs for infants who had high levels of *Bifidobacterium* in early life, accounting for an average of 38% of ARGs per sample detected in the year 2 samples. For infants who were low *Bifidobacterium* in early life, a mixed origin (meaning that the top hits from BLAST were from more than one family) was the most common predicted origin of ARGS, accounting for an average of 36% of ARGs per sample at year 2. In the year 2 samples from infants who were initially high *Bifidobacterium*, the detected families of origin in decreasing order of average per-sample origin were as follows: mixed, unknown (meaning either no BLAST hits or that the top three hits were all uncultured), *Streptococcaceae*, *Enterococcaceae*, *Pasteurellaceae*, *Prevotellaceae*, *Lactobacillaceae*, *Bifidobacteriaceae*, *Bacteroidaceae*, *Brachyspiraceae*, and *Campylobacteraceae*. In the year 2 samples from infants who were low *Bifidobacterium* in early life, the detected families of origin in decreasing order of average per-sample origin were as follows: *Enterobacteriaceae*, unknown, *Enterococcaceae*, *Streptococcaceae*, *Peptostreptococcaceae*, *Prevotellaceae*, *Bacteroidaceae*, *Bifidobacteriaceae*, *Pasteurellaceae*, *Clostridiaceae*, and *Pseudomonadaceae*.

**FIG 3:**
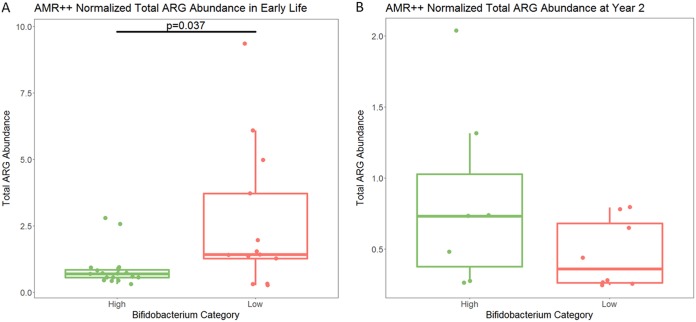
Total ARG reads normalized by read length and 16S gene read count in early life in Bangladesh. (A) Infants with high levels of *Bifidobacterium* had significantly lower levels of ARGs in their metagenomes (*P* = 0.037 by Kruskal-Wallis test) in early life. (B) At year 2, there was no significant difference in ARGs between infants who had high and low levels of *Bifidobacterium* in early life (*P* = 0.247 by Kruskal-Wallis). *P* value for significance is 0.05, as total ARG is the sum of all other ARGs that are compared later to identify particular differences.

The merged reads were then classified as ARGs using the AMR++ pipeline and normalized by the number of reads mapping to 16S rRNA genes to examine both transferable and nontransferable ARGs. Table S1 reports on the number of 16S reads identified by METAXA2 and the number of reads mapping to ARGs by AMR++. Normalization was completed to calculate what is referred to as abundance using the number of reads mapping to each ARG, the length of the full gene of each ARG, and the number of reads mapping to 16S rRNA genes, as described by Li et al. (35) Comparing the total numbers of normalized reads mapping to ARGs by AMR++ between high- and low-*Bifidobacterium* samples, high-*Bifidobacterium* samples had a significantly lower abundance of ARGs than low-*Bifidobacterium* samples (median for high *Bifidobacterium*, 0.69; median for low *Bifidobacterium*, 1.42; *P* = 0.037) (Fig. 3A). There was no significant difference in total ARGs by delivery mode in either the high-*Bifidobacterium* infants (*P* = 0.31) or the low-*Bifidobacterium* infants (*P* = 0.88).

Because the MEGARes database organizes ARGs into the acyclical categories of group, mechanism, and class (36), high- and low-*Bifidobacterium* samples were compared for differences at each of these levels as well as for differences in individual ARGs. There were a total of 18 distinct AMR classes detected in the early-life samples using AMR++, leading to a Bonferroni-corrected *P* value of 0.00278 to detect a significant difference in normalized ARG abundance in class between high- and low-*Bifidobacterium* samples using a Kruskal-Wallis test. Of these 18 classes, 10 were at higher normalized abundance levels in low-*Bifidobacterium* early-life samples: aminocoumarins (*P* = 0.000141), aminoglycosides (*P* = 0.000987), bacitracin (*P* = 0.000242), beta-lactams (*P* = 0.0000127), cationic antimicrobial peptides (*P* = 0.00186), elfamycins (*P* = 0.00000582), fluoroquinolones (*P* = 0.0000588), multidrug resistance (MDR) (*P* = 0.000122), rifampin (*P* = 0.0000151), and sulfonamides (*P* = 0.000181). In addition, the high-*Bifidobacterium* samples were significantly higher in a single class of AMR in early-life samples, MLS (*P* = 0.00000503). Because beta-lactam antibiotics are clinically important, to further explore the resistance to beta-lactam present in these samples, the 240 ARGs belonging to the class of beta-lactamases were classified as either transferable (229 ARGs) or nontransferable (11 ARGs) based on whether or not the gene was included in the ResFinder database, and the abundances of both transferable and nontransferable beta-lactamases were compared between high- and low-*Bifidobacterium* samples using a Kruskal-Wallis test. Both transferable and nontransferable beta-lactam ARGs were significantly increased in low-*Bifidobacterium* infants. For transferable beta-lactam ARGs, the median normalized abundance was 0.0941 in the low-*Bifidobacterium* early-life samples but only 0.00171 in the high-*Bifidobacterium* samples (*P* < 0.0001). For nontransferable beta-lactam ARGs, the median normalized abundance was 0.0505 for the low-*Bifidobacterium* samples but only 0.00286 for the high-*Bifidobacterium* early-life samples (*P* < 0.0001).

At the mechanism level, a total of 48 mechanisms were detected, resulting in a Bonferroni-corrected *P* value of 0.00104. A total of 13 of these mechanisms were significantly elevated in the low-*Bifidobacterium* early-life samples: aminocoumarin-resistant DNA topoisomerases (*P* = 0.000141), aminoglycoside efflux pumps (*P* = 0.000362), aminoglycoside efflux regulator (*P* = 0.000348), class A beta-lactamases (*P* = 0.0000229), EF-Tu inhibition (*P* = 0.00000582), fluoroquinolone-resistant DNA topoisomerases (*P* = 0.0000496), multidrug-resistant (MDR) mutant porin proteins (*P* = 0.0000320), MDR regulator (*P* = 0.0000874), multidrug efflux pumps (*P* = 0.000181), penicillin binding protein (*P* = 0.0000735), rifampin-resistant beta-subunit of RNA polymerase RpoB (*P* = 0.0000151), sulfonamide-resistant dihydropteroate synthases (*P* = 0.000181), and undecaprenyl pyrophosphate phosphatase (*P* = 0.000242). In high-*Bifidobacterium* early-life samples, only a single mechanism was significantly increased, 23S rRNA methyltransferases (*P* = 0.00000281); this mechanism belongs to class MLS. There were 178 different AMR groups (details on groups available on the MEGARes website, https://megares.meglab.org/browse/) detected in the early-life samples, resulting in a Bonferroni-corrected *P* value of 0.000281 for significance. Of these, 25 groups were significantly higher in the low-*Bifidobacterium* early-life samples: ACRB (*P* = 0.0000464), ACRD (*P* = 0.000248), AMPH (*P* = 0.0000796), ASMA (*P* = 0.000106), BACA (*P* = 0.000242), BAER (*P* = 0.000211), CPXA (*P* = 0.0000465), CPXAR (*P* =0.000110), CRP (*P* = 0.0000417), EMRB (*P* = 0.000110), ERMD (*P* = 0.000276), EMRK (*P* = 0.000170), EMRR (*P* = 0.000242), EVGA (*P* = 0.000182), FOLP (*P* = 0.000265), GYRA (*P* = 0.000164), GYRB (*P* = 0.0000602), HNS (*P* = 0.0000100), MARR (*P* = 0.000215), MDTB (*P* = 0.000248), OMPF (*P* = 0.0000320), PARC (*P* = 0.000226), PARE (*P* = 0.000101), RPOB (*P* = 0.0000151), and TUFAB (*P* = 0.00000582). There was also a single group of AMR elevated in the high-*Bifidobacterium* samples, ERMX (*P* = 0.00000279), belonging to mechanism 23S rRNA methyltransferases (Table 3 summarizes class, mechanism, and group results). There were a total of 724 different ARGs detected in the early-life samples, resulting in a Bonferroni correction for multiple comparisons of 0.0000691. As a result of this stringent correction, there were 6 genes significantly enriched in low-*Bifidobacterium* samples and 4 genes significantly enriched in high-*Bifidobacterium* samples (Table S3). All four genes enriched in the high-*Bifidobacterium* samples belonged to the group ERMX, and BLAST searching against the full reference sequence of the gene indicated that all four ERMX genes are known to occur in *Bifidobacterium*. All classes, mechanisms, groups, and genes that were significantly different between the high- and low-*Bifidobacterium* samples in the AMR++ analysis were present in at least 2 high-*Bifidobacterium* samples and at least 2 low-*Bifidobacterium* samples.

**TABLE 3:** Median normalized abundance for AMRs identified by MEGARes that were significantly different between the high- and low-*Bifidobacterium* samples in Bangladesh^*a*^

Database level and AMR class, mechanism, or group value	Median normalized abundance in samples (range) with:		Kruskal-Wallis test *P* value
	High *Bifidobacterium*	Low *Bifidobacterium*	
Class			
Aminocoumarins	0.000275 (0–0.00603)	0.0236 (0–0.0708)	0.000141
Aminoglycosides	0.00374 (0–0.147)	0.0768 (0.00526–0.785)	0.000987
Bacitracin	0 (0–0.0166)	0.0116 (0.000491–0.129)	0.000242
Beta-lactams	0.00437 (0–0.068)	0.221 (0.0162–1.33)	0.0000127
Cationic antimicrobial peptides	0 (0–0.0594)	0.0322 (0–0.736)	0.00186
Elfamycins	0.00325 (0–0.0181)	0.0870 (0.00977–0.231)	0.00000582
Fluoroquinolones	0.00136 (0–0.0430)	0.100 (0.00238–0.448)	0.0000588
Multidrug resistance (MDR)	0.00672 (0–0.319)	0.567 (0.0204–4.19)	0.000122
Rifampin	0.00241 (0–0.0114)	0.0920 (0.00233–0.134)	0.0000150
Sulfonamides	0.000658 (0–0.00883)	0.0273 (0.000373–0.290)	0.000181
Macrolides, lincosamides, and streptogramins (MLS)	0.367 (0.224–2.74)	0.0760 (0.000908–0.273)	0.00000503
Mechanism			
Aminocoumarin-resistant DNA topoisomerases (class aminocoumarins)	0.000275 (0–0.00603)	0.0236 (0–0.0708)	0.000141
Aminoglycoside efflux pumps (class aminoglycosides)	0.000124 (0–0.0266)	0.0251 (0.00184–0.371)	0.000362
Aminoglycoside efflux regulator (class aminoglycosides)	0 (0–0.0166)	0.00577 (0–0.147)	0.000348
Class A beta-lactamases (class beta-lactamases)	0 (0–0.0455)	0.0591 (0.00160–0.725)	0.0000229
EF-Tu inhibition (class elfamycins)	0.00325 (0–0.018)	0.0870 (0.00977–0.231)	0.00000582
Fluoroquinolone-resistant DNA topoisomerases (class fluoroquinolones)	0.00136 (0–0.0430)	0.0729 (0.00238–0.447)	0.0000496
MDR mutant porin proteins (class MDR)	0.000356 (0–0.00686)	0.0227 (0.000493–0.105)	0.0000320
MDR regulator (class MDR)	0.00400 (0–0.137)	0.244 (0.0109–1.54)	0.0000874
Multidrug efflux pumps (class MDR)	0.00237 (0–0.194)	0.293 (0.00892–2.56)	0.000181
Penicillin binding protein (class beta-lactams)	0.00354 (0–0.0184)	0.0819 (0.00699–0.345)	0.0000735
Rifampin-resistant beta-subunit of RNA polymerase RpoB (class rifampin)	0.00241 (0–0.0114)	0.0920 (0.00233–0.134)	0.0000150
Sulfonamide-resistant dihydropteroate synthases (class sulfonamides)	0.000658 (0–0.00883)	0.0273 (0.000373–0.290)	0.000181
Undecaprenyl pyrophosphate phosphatase (class bacitracin)	0 (0–0.0166)	0.0116 (0.000491–0.129)	0.000242
23S rRNA methyltransferases (class MLS)	0.366 (0.220–2.72)	0.0140 (0–0.203)	0.00000281
Group			
ACRB (class MDR)	0 (0–0.00455)	0.0141 (0–0.0836)	0.0000464
ACRD (class aminoglycoside)	0 (0–0.0117)	0.0110 (0.000619–0.114)	0.000248
AMPH (class beta-lactam)	0.000454 (0–0.00872)	0.0176 (0.00131–0.123)	0.0000796
ASMA (class MDR)	0.000312 (0–0.00944)	0.0163 (0.000575–0.108)	0.000106
BACA (class bacitracin)	0 (0–0.0166)	0.0116 (0.000491–0.129)	0.000242
BAER (class MDR)	0 (0–0.00932)	0.0111 (0.000280–0.123)	0.000211
CPXA (class MDR)	0 (0–0.00772)	0.00856 (0–0.0814)	0.0000465
CPXAR (class MDR)	0 (0–0.0178)	0.0265 (0.000554–0.231)	0.000110
CRP (class MDR)	0.000726 (0–0.0105)	0.0236 (0.00144–0.132)	0.0000417
EMRB (class MDR)	0 (0–0.0124)	0.0158 (0.000659–0.131)	0.000110
EMRD (class MDR)	0 (0–0.00411)	0.00416 (0–0.0436)	0.000276
EMRK (class MDR)	0 (0–0.00802)	0.00534 (0–0.107)	0.000170
EMRR (class MDR)	0 (0–0.0129)	0.00909 (0.00114–0.104)	0.000242
EVGA (class MDR)	0 (0–0.0101)	0.00597 (0–0.113)	0.000182
FOLP (class sulfonamides)	0.000517 (0–0.00800)	0.0273 (0–0.149)	0.000265
GYRA (class fluoroquinolones)	0.000342 (0–0.00969)	0.0256 (0.000463–0.110)	0.000164
GYRB (class fluoroquinolones)	0.000230 (0–0.0145)	0.0225 (0.00132–0.144)	0.0000602
HNS (class MDR)	0.000179 (0–0.00849)	0.0152 (0.00147–0.0966)	0.0000100
MARR (class MDR)	0 (0–0.00611)	0.00428 (0–0.0902)	0.000215
MDTB (class MDR)	0 (0–0.0120)	0.0142 (0.000601–0.109)	0.000248
OMPF (class MDR)	0.000356 (0–0.00686)	0.0163 (0.000493–0.0930)	0.0000320
PARC (class fluoroquinolones)	0.000544 (0–0.0134)	0.0197 (0–0.132)	0.000226
PARE (class aminocoumarins)	0.000275 (0–0.00603)	0.0228 (0–0.0708)	0.000101
RPOB (class rifampin)	0.00241 (0–0.0114)	0.0920 (0.00233–0.134)	0.0000150
TUFAB (class elfamycin)	0.00325 (0–0.0181)	0.0870 (0.00977–0.230)	0.00000582
ERMX (class MLS)	0.366 (0.220–2.72)	0.0109 (0–0.0450)	0.00000279

a *P* value for significant difference at the class level was 0.00278, at the mechanism level was 0.00104, and at the group level was 0.000281.

10.1128/mSphere.00441-18.8 TABLE S3 Significantly different ARGs, groups, mechanisms, and classes using Kruskal-Wallis test after Bonferroni correction. Bold black text indicates enrichment in low-*Bifidobacterium* samples; bold red text indicates significant enrichment in high-*Bifidobacterium* samples. Please see text and Table 3 for *P* values for significance. Download Table S3, PDF file, 0.1 MB. Copyright © 2018 Taft et al. 2018 Taft et al. This content is distributed under the terms of the Creative Commons Attribution 4.0 International license.

To confirm that the AMR++ results were not an artifact of the method, early-life samples were also analyzed using ShortBRED with the Comprehensive Antibiotic Resistance Database (CARD) 2017 pregenerated ShortBRED database (37, 38). Because CARD does not have the acyclical classification scheme of MEGARes, only total counts and individual gene counts were compared between high- and low-*Bifidobacterium* samples. In addition, the number of ARGs included in CARD is smaller than the number of ARGs included in MEGARes, which will reduce the number of ARGs detected in the early-life samples. Results were generally similar to AMR++ results with a significantly lower total AMR value in high-*Bifidobacterium* samples than in low-*Bifidobacterium* samples (Kruskal-Wallis test, *P* = 0.0014) (Fig. S4). ShortBRED identified a total of 237 ARGs in the early-life samples, resulting in a Bonferroni-corrected *P* value of 0.000211 for significance. There were a total of 14 genes that were significantly reduced in the high-*Bifidobacterium* samples using a Kruskal-Wallis test, including ARO3002985 (cationic antimicrobial peptide resistance, *P* = 0.000147), ARO3003369 (elfamycin resistance, *P* = 0.0000454), ARO3004042 (MDR, *P* = 0.000143), ARO3000518 (MDR, *P* = 0.0000153), ARO3003890 (fosfomycin, *P* = 0.0000735), ARO3000832 (MDR, *P* = 0.0000531), ARO3003288 (rifampin, *P* = 0.00000991), ARO3003807 (MDR, *P* = 0.000180), ARO3003950 (nitroimidazole, *P* = 0.000110), ARO3002818 (MLS, *P* = 0.000161), ARO3003511 (MDR, *P* = 0.000173), ARO3000826 (MDR, *P* = 0.0000159), ARO3003317 (fluoroquinolone, *P* = 0.000130), and ARO3000263 (MDR, *P* = 0.000111). There was a single gene that was significantly enriched in the high-*Bifidobacterium* samples using a Kruskal-Wallis test, ARO3000596 (MLS, *P* = 0.00000282). More genes were identified as significantly different by ShortBRED than by AMR++, likely resulting from the smaller size of CARD, meaning that a less stringent Bonferroni correction was applied to the ShortBRED analysis. Both ShortBRED and AMR++ identified increased ErmX resistance in the high-*Bifidobacterium* samples. Unlike AMR++, ShortBRED did not identify any beta-lactamases that were significantly enriched in the low-*Bifidobacterium* samples; however, the specific gene identified as enriched by AMR++ was not included in CARD. ShortBRED identified significant differences in a gene providing resistance to fosfomycin and a gene providing resistance to nitroimidazole, where AMR++ did not identify any differences in resistance to either of these resistance classes; this may relate to the more stringent *P* value correction used in the AMR++ analysis.

10.1128/mSphere.00441-18.4 FIG S4 Total AMR count in early-life samples as measured by ShortBRED. High-*Bifidobacterium* samples had significantly fewer ARGs than low-*Bifidobacterium* samples (*P* = 0.0014, Kruskal-Wallis test). A *P* value of 0.05 was used for significance on this test. Download FIG S4, PDF file, 0.1 MB. Copyright © 2018 Taft et al. 2018 Taft et al. This content is distributed under the terms of the Creative Commons Attribution 4.0 International license.

In the year 2 samples, there was no significant difference in the number of AMR classes or ARGs detected by ResFinder (*P* = 0.725 and *P* = 0.685, respectively; Kruskal-Wallis test). For the AMR++ pipeline at year 2, only ARGs, groups, mechanisms, and classes that were significantly different in early life were compared. There were no significant differences at year 2 in total normalized ARGs between infants who had high *Bifidobacterium* and infants who had low *Bifidobacterium* in early life (*P* = 0.247, Kruskal-Wallis test). Only the ARGs, groups, mechanisms, and classes that were significantly different in early life were tested in later life to minimize multiple-comparison issues. Despite this, there were no remaining significant differences in any specific ARG, group, mechanism, or class at year 2.

**FIG 4:**
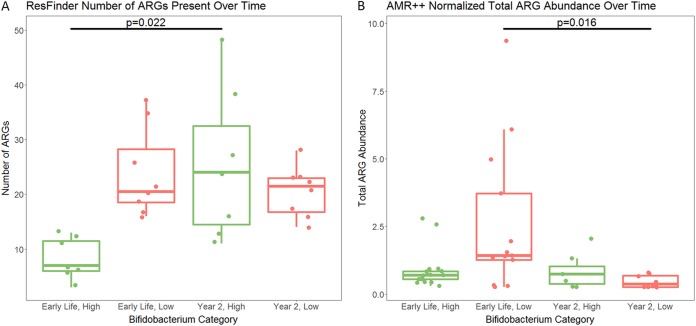
Comparison between early-life and year 2 AMR levels. (A) There was a significant increase in the number of transferable ARGs present (as detected by ResFinder) in the high-*Bifidobacterium* infants between early life and year 2 (*P* = 0.0223, Wilcoxon paired test). There was no significant difference in the number of transferable ARGs present in low-*Bifidobacterium* infants between the early-life and year 2 samples (*P* = 0.324, Wilcoxon paired test). (B) There was no significant difference in AMR abundance between early life and year 2 in infants who had high levels of *Bifidobacterium* in early life (*P* = 0.938, Wilcoxon paired test). There was a significant decrease in AMR abundance between early life and year 2 in infants who had low levels of *Bifidobacterium* in early life (*P* = 0.0156, Wilcoxon paired test). *P* value for significance is 0.05.

Comparing early-life samples with year 2 samples, there was no significant difference in the number of AMR classes or ARGs detected by ResFinder in the low-*Bifidobacterium* samples (*P* = 0.672 and *P* = 0.324, respectively; Wilcoxon paired test). However, there was a significant increase in the number of both AMR classes and ARGs in the high-*Bifidobacterium* samples (*P* = 0.022 and *P* = 0.022, respectively; Wilcoxon paired test). The AMR++ pipeline had contrasting results: there was no significant difference in AMR abundance between early-life and year 2 samples for high-*Bifidobacterium* infants (Wilcoxon paired test, *P* = 0.938). In contrast, there was a significant drop in AMR abundance between early-life and year 2 infants with low levels of *Bifidobacterium* in early life (Wilcoxon paired test, *P* = 0.0156) (Fig. 4).

To further confirm that levels of *Bifidobacterium* were different between the high- and low-*Bifidobacterium* samples, quantitative PCR (qPCR) was conducted to measure total bacteria, total *Bifidobacterium*, and total *Enterobacteriaceae* in all samples where sufficient DNA remained after sequencing. The *Enterobacteriaceae* family was chosen for qPCR because it was the most common predicted origin of AMR in the ResFinder analysis. This analysis included 28 of the 31 early-life samples and 14 of the 15 year 2 samples. In early life, the high-*Bifidobacterium* samples had significantly lower total bacteria than the low-*Bifidobacterium* samples (Kruskal-Wallis test, *P* = 0.00402) (Fig. S5A), but in later life there was no significant difference in total bacteria between these groups (Kruskal-Wallis test, *P* = 0.338) (Fig. S5A). The infants with early-life high *Bifidobacterium* exhibited a significant increase in total bacteria between the early-life and year 2 samples (Wilcoxon paired test, *P* = 0.0156), but there was no significant difference in total bacteria between early life and year 2 in the early-life low-*Bifidobacterium* infants (Wilcoxon paired test, *P* = 0.312). However, the total *Bifidobacterium* qPCR confirmed that in early life the high-*Bifidobacterium* samples had significantly more *Bifidobacterium* than the low-*Bifidobacterium* samples (Kruskal-Wallis test, *P* < 0.0001) (Fig. S5B). There was no significant difference in *Bifidobacterium* levels between the two groups at year 2 (Kruskal-Wallis test, *P* = 0.565). While not reaching significance, there was a trend toward a reduction in *Bifidobacterium* levels in the high-*Bifidobacterium* group when comparing early-life and year 2 samples (Wilcoxon paired test, *P* = 0.0781). There was also a trend toward increased *Bifidobacterium* levels in infants in the low-*Bifidobacterium* group between early life and year 2 (Wilcoxon paired test, *P* = 0.0625). The *Enterobacteriaceae* qPCR found that high-*Bifidobacterium* infants in early life had significantly lower *Enterobacteriaceae* than low-*Bifidobacterium* infants (Kruskal-Wallis test, *P* = 0.00961) (Fig. S5C). By year 2, the difference in *Enterobacteriaceae* between the high- and low-*Bifidobacterium* groups was only borderline significant (Kruskal-Wallis test, *P* = 0.0693). There was no significant difference in *Enterobacteriaceae* between the early-life and year 2 samples in the infants with low *Bifidobacterium* in early life (Wilcoxon paired test, *P* = 0.812), but there was a significant increase in *Enterobacteriaceae* in the high-*Bifidobacterium* infants between early life and year 2 (Wilcoxon paired test, *P* = 0.0156). The lower total bacteria and *Enterobacteriaceae* in the samples with higher levels of *Bifidobacterium* are consistent with our hypothesis that acid production by *Bifidobacterium* suppresses the growth of other taxa, potentially allowing niches that might otherwise be filled by AMR-carrying bacteria to remain open during infancy. The qPCR results for total *Bifidobacterium* levels were significantly correlated with the *Bifidobacterium* relative abundance calculated from the 16S rRNA gene sequencing data (Spearman’s rho = 0.82, *P* < 0.0001) and with the *Bifidobacterium* relative abundance calculated from the WGS data (Spearman’s rho = 0.73, *P* < 0.0001). The correlation becomes stronger after dividing the *Bifidobacterium* qPCR results by the total bacterial qPCR results (with 16S rRNA gene sequencing data, rho = 0.86, *P* < 0.0001, and with WGS data, rho = 0.78, *P* < 0.0001). The value of the relative abundance calculated from the qPCR data is potentially problematic, as 10 of the 18 high-*Bifidobacterium* samples were predicted to have a *Bifidobacterium* relative abundance greater than 100% and 3 samples were predicted to have a *Bifidobacterium* relative abundance greater than 200%. This may be related to the use of different 16S regions for the two sets of primers. In addition, higher total *Bifidobacterium* levels in early life correlated with reduced total normalized AMR abundance (from AMR++ results, Spearman’s test, rho = −0.500, *P* = 0.00736), but there was no significant correlation between either *Enterobacteriaceae* and total normalized AMR abundance (Spearman’s test, *P* = 0.134) or total bacteria and total normalized AMR abundance (Spearman’s test, *P* = 0.601).

10.1128/mSphere.00441-18.5 FIG S5 qPCR results. (A) Log-transformed concentration of total bacteria (CFU per milliliter). Low-*Bifidobacterium* samples have significantly more total bacteria than high-*Bifidobacterium* samples in early life (Kruskal-Wallis test, *P* = 0.00402). There was no significant difference in total bacteria between the early-life high-*Bifidobacterium* and the early-life low-*Bifidobacterium* infants at year 2 (Kruskal-Wallis test, *P* = 0.338). The early-life high-*Bifidobacterium* infants experienced a significant increase in total bacteria from early life to year 2 (Wilcoxon paired test, *P* = 0.0156). There was no significant difference in total bacteria between the early and year 2 samples for early-life low-*Bifidobacterium* infants (Wilcoxon paired test, *P* = 0.312). (B) Log-transformed concentration of *Bifidobacterium* (CFU per milliliter). Early-life high-*Bifidobacterium* samples have significantly more *Bifidobacterium* than early-life low-*Bifidobacterium* samples (Kruskal-Wallis test, *P* < 0.0001). However, there was no significant difference in *Bifidobacterium* levels at year 2 between infants who were high *Bifidobacterium* in early life and those who were low *Bifidobacterium* in early life (Kruskal-Wallis test, *P* = 0.565). Comparing high-*Bifidobacterium* samples with both an early-life and a year 2 sample, there was a trend toward lower levels of *Bifidobacterium* at year 2 (Wilcoxon paired test, *P* = 0.0781). Comparing low *Bifidobacterium* samples with both an early-life and a year 2 sample, there was a trend toward higher levels of *Bifidobacterium* in the year 2 samples (Wilcoxon paired test, *P* = 0.0625). (C) Log-transformed concentration of *Enterobacteriaceae* (CFU per milliliter). Early-life high-*Bifidobacterium* samples have significantly less *Enterobacteriaceae* than early-life low-*Bifidobacterium* samples (Kruskal-Wallis test, *P* = 0.00961). However, there was no significant difference in *Enterobacteriaceae* levels at year 2 between infants who were high *Bifidobacterium* in early life and those who were low *Bifidobacterium* in early life (Kruskal-Wallis test, *P* = 0.0693). There was no significant change in *Enterobacteriaceae* levels between early life and year 2 in low *Bifidobacterium* infants (Wilcoxon paired test, *P* = 0.812), but there was a significant increase in *Enterobacteriaceae* levels between early life and year 2 in high Bifidobacterium infants (Wilcoxon paired test, *P* = 0.0156). A *P* value of 0.0167 was used for significance. Download FIG S5, PDF file, 0.2 MB. Copyright © 2018 Taft et al. 2018 Taft et al. This content is distributed under the terms of the Creative Commons Attribution 4.0 International license.

### Additional cohort analysis: infants from Sweden.

To confirm the findings of AMR reduction detected in the Bangladeshi cohort, the month 4 and month 12 samples from the study by Bäckhed et al. (30) of infant microbial colonization dynamics of Swedish infants were analyzed using the AMR++ pipeline. The ResFinder analysis was not completed in the Swedish cohort because at month 4 higher levels of *Bifidobacterium* correlate with a lower trimmed read depth (Spearman’s test, *P* < 0.0001), which will bias the results away from the null hypothesis. There was no significant correlation between *Bifidobacterium* and read depth at month 12 (Spearman’s test, *P* = 0.206). The AMR++ pipeline analysis does include a normalization step that adjusts for read depth. Table S4 presents the read depth of downloaded sequence files, host-subtracted sequence files, trimmed reads, number of 16S reads by METAXA2, and number of reads mapping to ARGs by AMR++. To first explore the connection between *Bifidobacterium* levels and total AMR including all infants at month 4, total AMR was log transformed. A regression model was then run with the log-transformed total AMR as the outcome variable and the *Bifidobacterium* level in the sample, the number of times that an infant received antibiotics before age 4 months, and delivery mode as predictor variables using the glm command in R 3.4.3. Relative abundance of *Bifidobacterium* in the sample was significantly associated with the log-transformed total AMR (*P* = 0.00785, β = −1.5654), while the number of times that an infant received antibiotics (*P* = 0.657) and delivery mode (*P* = 0.24) were nonsignificant. The association between *Bifidobacterium* and the log of the total AMR remained significant after removing antibiotic exposure and delivery mode from the model (*P* = 0.0159, β = −1.3886). This is consistent with increasing levels of *Bifidobacterium* correlating with decreasing levels of AMR. However, as only 5 infants of the 100 Swedish infants ever received antibiotics, power may be limited to detect the actual influence of antibiotic exposure on AMR level.

10.1128/mSphere.00441-18.9 TABLE S4 Swedish cohort sequencing results. Because of the larger insert size, reads were not merged. Download Table S4, PDF file, 0.2 MB. Copyright © 2018 Taft et al. 2018 Taft et al. This content is distributed under the terms of the Creative Commons Attribution 4.0 International license.

**FIG 5:**
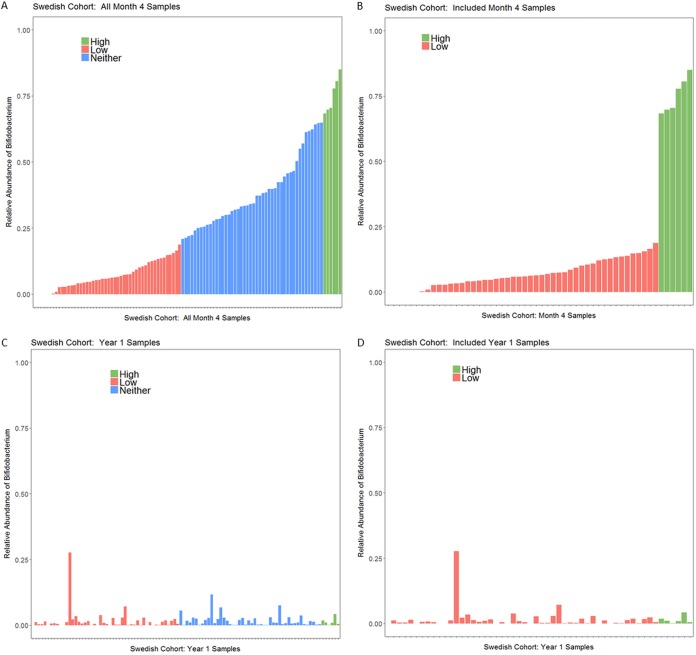
Relative abundance of *Bifidobacterium* of Swedish infants at month 4. (A) *Bifidobacterium* relative abundance in all month 4 samples. (B) *Bifidobacterium* relative abundance at month 4 in only samples included in the AMR analysis. The empty space at the start of bar charts in panels A and B is for the 6 samples that had no or almost no *Bifidobacterium* present. (C) *Bifidobacterium* levels at year 1; samples are in the same order as panel A and are color coded by *Bifidobacterium* levels at month 4. (D) Plot showing the year 1 samples from only infants classified as either high or low *Bifidobacterium* at month 4; these were the infants included in the year 1 analyses.

**FIG 6:**
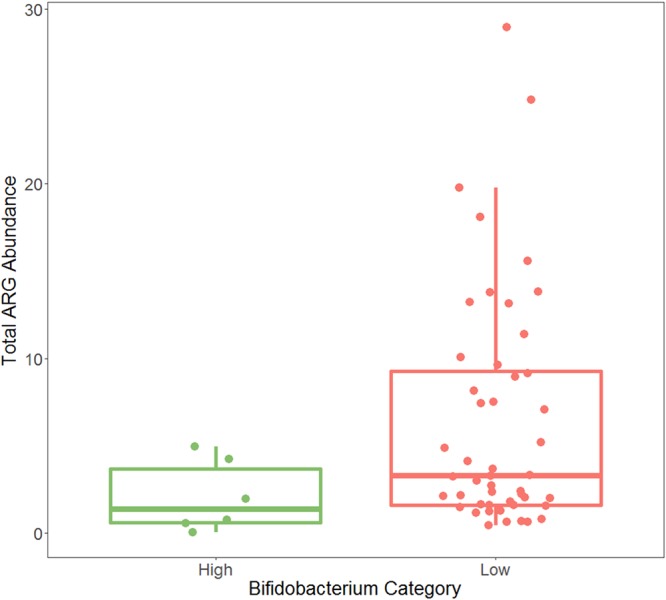
Total ARG reads normalized by read length and 16S read count at month 4 in the Swedish infants. There was no significant difference in total ARG abundance between high- and low-*Bifidobacterium* infants (Kruskal-Wallis test, *P* = 0.0613). *P* value for significance is 0.05.

Of the 100 infants at month 4, only 6 met the criteria for high *Bifidobacterium* while 48 met the criteria for low *Bifidobacterium* (Fig. 5). At month 4, there was no significant difference in the total abundances of ARGs detected in high- and low-*Bifidobacterium* samples (median for high *Bifidobacterium*, 1.35; for low *Bifidobacterium*, 3.27; *P* = 0.0613) (Fig. 6). In this cohort, there were 17 different AMR classes detected, resulting in a Bonferroni-corrected *P* value of 0.00294 for a significant difference in class abundance at month 4. Only one of the seventeen classes differed significantly between high- and low-*Bifidobacterium* infants, tetracyclines (Kruskal-Wallis test, *P* = 0.000294), with low-*Bifidobacterium* infants having significantly higher levels of tetracycline resistance.

There were a total of 47 AMR mechanisms detected in the month 4 samples classified as high or low *Bifidobacterium*, resulting in a Bonferroni-corrected *P* value of 0.00106 for comparisons. Only two mechanisms were significantly enriched in month 4 low-*Bifidobacterium* samples: class A beta-lactamases (*P* = 0.000347) and tetracycline resistance ribosomal protection proteins (*P* = 0.000153). No AMR mechanisms were significantly enriched in high-*Bifidobacterium* samples. There were 173 different AMR groups present in the month 4 high- or low-*Bifidobacterium* samples, resulting in a Bonferroni-corrected *P* value of 0.000289 for significance. There was one group significantly increased in the low-*Bifidobacterium* samples, TETQ (*P* = 0.0000467). There were 599 different ARGs detected in the month 4 high- and low-*Bifidobacterium* samples, resulting in a Bonferroni-corrected *P* value of 0.0000835. No individual ARGs differed significantly between the high- and low-*Bifidobacterium* month 4 samples.

**FIG 7:**
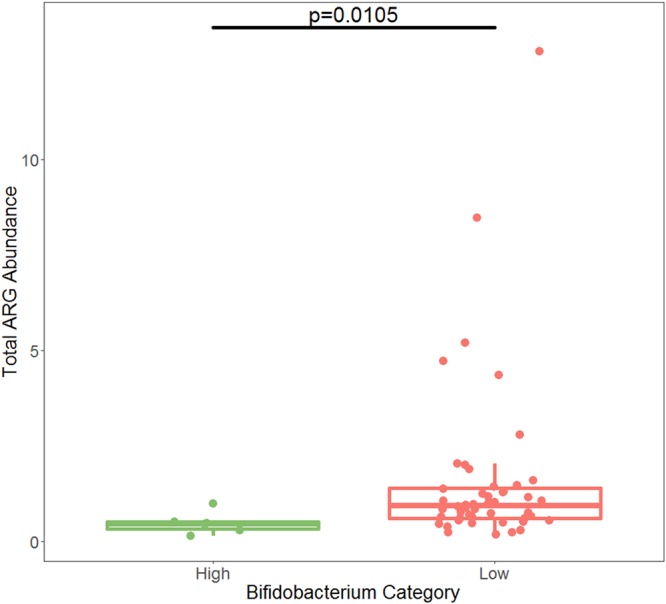
Total ARG abundance at year 1 in the Swedish cohort. Samples from infants with low *Bifidobacterium* at month 4 had significantly more ARG abundance than samples from infants who had high *Bifidobacterium* at month 4 (Kruskal-Wallis test, *P* = 0.0105). *P* value for significance is 0.05.

**FIG 8:**
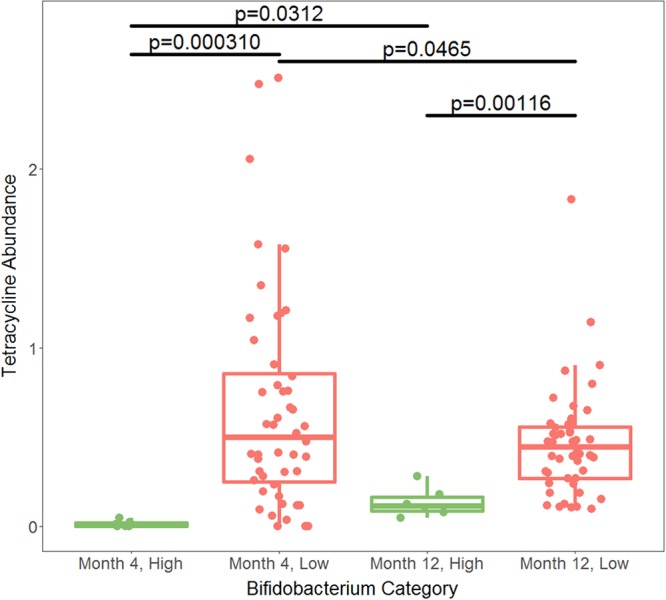
Normalized abundance of tetracycline resistance genes in Swedish infants by AMR++. High-*Bifidobacterium* infants had significantly less tetracycline resistance at both month 4 (*P* = 0.000310, Kruskal-Wallis test) and year 1 (*P* = 0.00116, Kruskal-Wallis test). This is despite an increase in tetracycline resistance in the high-*Bifidobacterium* infants (*P* = 0.0312, Wilcoxon paired test) and a decrease in tetracycline resistance in the low-*Bifidobacterium* infants (*P* = 0.00116, Wilcoxon paired test). As tetracycline was the only class of AMR to meet the *P* value of 0.00294 for significance, it was the only class compared at the later time points. This means that comparisons using the year 1 data had a *P* value of 0.05 for significance.

By month 12, the relative abundance of *Bifidobacterium* for all infants had fallen below 30%, and below 20% for all infants except one (Fig. 5C). The one infant with *Bifidobacterium* abundance above 20% at month 12 was an infant with *Bifidobacterium* abundance below 20% at month 4, which suggests that infants in the Swedish cohort may have had variable levels of *Bifidobacterium*, as was observed in the Bangladeshi cohorts. The Swedish infants had much lower rates of breastfeeding at 1 year than the breastfeeding rates typical of Bangladesh. Only 14 of 98 infants with information on feeding at month 12 received any breast milk in their diet, which may explain why children in Sweden tended to have a lower prevalence of *Bifidobacterium* at month 12 than children in Bangladesh at year 2. At month 12, infants who had low levels of *Bifidobacterium* at month 4 had significantly higher total ARG abundance than infants with high levels of *Bifidobacterium* at month 4 (*P* = 0.0105) (Fig. 7). Because the only significant difference in AMR classes between high and low *Bifidobacterium* was in tetracycline, only this class was tested at month 12, using a *P* value of 0.05. Infants who had high levels of *Bifidobacterium* at month 4 had a significantly lower abundance of reads belonging to class tetracycline than infants who had low levels of *Bifidobacterium* at month 4 when comparing infant microbiomes at month 12 (Kruskal-Wallis test, *P* = 0.00116), despite an increase in tetracycline abundance in the high-*Bifidobacterium* samples between months 4 and 12 (Wilcoxon paired test, *P* = 0.0312) and a decrease in tetracycline abundance in the low-*Bifidobacterium* samples between months 4 and 12 (Wilcoxon paired test, *P* = 0.0465) (Fig. 8). Only the two mechanisms that differed significantly at 4 months were tested at 12 months, with a Bonferroni-corrected *P* value for significance of 0.025. There was no significant difference in the mechanism class A beta-lactamases (*P* = 0.159); however, month 4 low-*Bifidobacterium* infants had a significantly higher abundance of the mechanism tetracycline resistance ribosomal protection proteins than month 4 high-*Bifidobacterium* infants (*P* < 0.0001).

**FIG 9:**
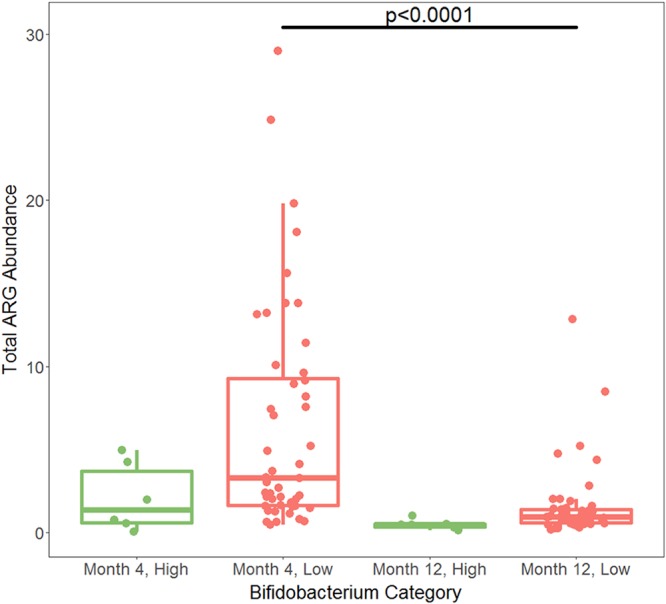
AMR++ normalized total ARG abundance over time. Comparison between month 4 and year 1 AMR levels in the Swedish cohort. There was no significant difference in AMR abundance between the month 4 and year 1 high-*Bifidobacterium* samples (*P* = 0.156, Wilcoxon paired test). There was a significant decrease in AMR abundance between the month 4 and year 1 low-*Bifidobacterium* samples (*P* < 0.0001, Wilcoxon paired test). Significance was determined using a *P* value of 0.05.

Comparing levels of AMR in infants at month 4 with those in infants at month 12, high-*Bifidobacterium* infants did not have significantly different abundances of total AMR (*P* = 0.156, Wilcoxon paired test). However, as with the Bangladeshi cohort, low-*Bifidobacterium* early-life infants did experience a significant drop in the abundance of AMR between month 4 and month 12 (*P* < 0.0001, Wilcoxon paired test) (Fig. 9).

## DISCUSSION

AMR is a major public health challenge, and the gut microbiome may come to harbor ARGs even in the first few days of life (28, 29). Globally, there are an estimated 214,000 neonatal sepsis deaths each year attributable to AMR pathogens (39). In particular, resistance to the first-line antibiotics for sepsis, penicillin-ampicillin with gentamicin, is present in as many as 35% of older infants with sepsis and resistance to second-line cephalosporins is also common (40). In early life, *Bifidobacterium* at levels greater than 65% correlates strongly with reduced levels of ARGs, in terms of both a reduced diversity of transferable ARGs present and reduced ARG abundance in the Bangladeshi infants. The relative abundance differences in *Bifidobacterium* do correlate with differences in absolute abundance of *Bifidobacterium* by qPCR. In addition, the high-*Bifidobacterium* infants had lower levels of total bacteria and of family *Enterobacteriaceae* than the low-*Bifidobacterium* infants. Despite being the most common predicted origin of AMR, levels of family *Enterobacteriaceae* measured by qPCR did not correlate with a reduction in total AMR abundance in early life. This may be related to the small sample size of this analysis (only 28 samples had sufficient DNA for qPCR). That there was a significant correlation between *Bifidobacterium* and a reduction in total AMR abundance suggests that *Bifidobacterium* may suppress more AMR-carrying taxa than just *Enterobacteriaceae*. This is consistent with past research that finds that *Bifidobacterium* has an inhibitory effect on the growth of a variety of other commensals, including pathogens, via organic acid (particularly acetate) and bacteriocin production (41–43). This potentially keeps niches that might otherwise be occupied by AMR-containing organisms open until weaning. In the Swedish infants, there was a trend toward a significant reduction in ARGs in the high-*Bifidobacterium* infants (*P* = 0.06). A key difference is that in the Swedish cohort the typical relative abundance of *Bifidobacterium* in early life was much lower, resulting in very few infants in the high category for the comparisons. However, a regression model testing for the association between total ARG abundance and *Bifidobacterium* level using all infants in the Swedish cohort did find a significant association, where higher levels of *Bifidobacterium* correlated with lower levels of total ARG abundance. This suggests that infants with gut microbiomes dominated by *Bifidobacterium* are at lower risk of AMR-associated diseases, although additional studies are needed to confirm this hypothesis.

Historically, the infant microbiome was dominated by *Bifidobacterium* (44, 45). However, by the 1970s in the United States, urban infants were beginning to exhibit a drop in *Bifidobacterium* levels in infant stool (46). Others have previously noted differential distribution of *Bifidobacterium* within neonates from different locations (8), suggesting that this benefit of AMR reduction by high early *Bifidobacterium* may be regional. *Bifidobacterium* is linked to a variety of beneficial health effects, including immunological modulations (9, 47) and improvement in barrier function (48, 49), among others. A recent intervention study showed that a high level of *Bifidobacterium* from supplementation with B. longum subsp. *infantis* correlates with lower fecal endotoxin (5). The high level of *Bifidobacterium* present in this Bangladeshi cohort was previously associated with improved vaccine response (9), and follow-on work has recently demonstrated that high early *Bifidobacterium* (in this case primarily B. longum subsp. *infantis*) correlates with improved vaccine responses at 2 years (M. N. Huda, S. M. Ahmad, M. J. Alam, A. Khanam, K. M. Kalanetra, D. H. Taft, R. Raqib, M. A. Underwood, D. A. Mills, and C. B. Stephensen, submitted for publication).

Of particular importance, high *Bifidobacterium* levels are associated with reduced beta-lactam resistance in early-life samples in Bangladesh. Beta-lactams such as amoxicillin are the first-line antimicrobials for acute otitis media (50). Importantly, high levels of *Bifidobacterium* also correlated with reduced transferable beta-lactam resistance in this cohort. In the Bangladeshi infants, the median level of transferable beta-lactam resistance was 55 times greater during early life in low-*Bifidobacterium* infants than in high-*Bifidobacterium* infants. While in the Swedish infants there was not a significant association between the abundance of all beta-lactams and *Bifidobacterium* levels, there was an association between reduced levels of class A beta-lactamases and high *Bifidobacterium* levels. This suggests that a microbiome dominated by *Bifidobacterium* not only has the potential to help protect infants at high risk of sepsis from disease caused by an AMR-bearing microorganisms generally but also that high *Bifidobacterium* levels may lower the level of the ARGs most likely to cause problems during treatment of sepsis. Supplementing breastfed infants with probiotics to dominate the gut with *Bifidobacterium* is experimentally possible; a recent study established that supplementing healthy breastfed term infants with *Bifidobacterium* in early life was well tolerated (4) and resulted in dramatically greater levels of *Bifidobacterium* in infant stool than those for nonsupplemented infants (5). This strategy may prove particularly effective in reducing AMR in populations where infants do not naturally acquire high levels of *Bifidobacterium*.

While high bifidobacterial levels correlated with reduced AMR during early life, there were mixed results in the effect of *Bifidobacterium* levels in the later-life samples. In the Bangladeshi samples, there was no association between early-life *Bifidobacterium* levels and AMR at age 2 years. Furthermore, the number of different transferable AMR classes and ARGs increased in the high-*Bifidobacterium* infants, despite no change in AMR abundance. The number of different transferable AMR classes and ARGs did not change in the low-*Bifidobacterium* infants. Together, this suggests that high levels of *Bifidobacterium* delayed the acquisition of ARGs and that the increasing levels of bacteria diluted the abundance of ARGs at later time points. In the Swedish cohort at year 1, the month 4 low-*Bifidobacterium* infants had significantly higher levels of ARGs than the month 4 high-*Bifidobacterium* infants. These same infants had a persistent reduction in tetracycline resistance levels at month 12 if they had high levels of *Bifidobacterium* at month 4. This may also reflect the shorter time period to acquire additional ARGs available to the Swedish cohort than the Bangladeshi cohort. In both the Bangladeshi infants and the Swedish infants, AMR abundance did not change significantly between early-life and later-life samples in infants with high levels of *Bifidobacterium* in early life. However, infants in both cohorts with low levels of early-life *Bifidobacterium* experienced a significant drop in AMR abundance between early-life and later samples. The discrepancy between the Bangladeshi and Swedish infants in whether high *Bifidobacterium* in early life is associated with a sustained reduction in at least one class of AMR highlights the need for more research into the biogeography and timing of infant colonization by AMR-carrying organisms. This study is limited by the availability of samples at different time points for inclusion; only a single early-life sample and a single sample from year 1 (Swedish infants) or from year 2 (Bangladeshi infants) were available, and the earlier timing of the Swedish samples may explain the discrepancy in whether differences in later-life total ARG abundance could be detected by early-life *Bifidobacterium* levels. The limited time points of collection in both the Swedish and Bangladeshi cohorts do not provide the data needed to fully describe the trajectory of AMR colonization in these infants or to explore how other environmental factors affect AMR colonization. An additional limitation of this study is a lack of data on antimicrobial use in the Bangladeshi cohort. It is possible that the infants with low *Bifidobacterium* received antimicrobials, which may drive an increase in AMR in the microbiome. To fully understand the relationship between *Bifidobacterium* dominance in infancy and AMR levels in early life, studies with dense sampling during infancy and childhood including infants from both developed and developing countries are needed to first understand how stability or fluctuations of *Bifidobacterium* dominance affect AMR levels and then to understand how far into the weaning process and under what environmental conditions a reduction in AMR associated with early high levels of *Bifidobacterium* may extend.

## MATERIALS AND METHODS

### Bangladeshi cohort.

This study used samples collected from infants enrolled in a clinical trial of vitamin A supplementation and vaccine effectiveness (ClinicalTrials.gov NCT01583972) and a follow-up study on *Bifidobacterium* levels in early infancy and vaccine response in early infancy and at age 2 years (NTC02027610) (26; M. N. Huda, S. M. Ahmad, M. J. Alam, A. Khanam, K. M. Kalanetra, D. H. Taft, R. Raqib, M. A. Underwood, D. A. Mills, and C. B. Stephensen, submitted for publication). Ethical approval was obtained from the Research Review Committee and the Ethical Review Committee of the International Centre for Diarrheal Disease Research, Bangladesh. Stool samples were collected from 291 infants at age weeks 6, 11, and 15, and 250 of these infants also provided a sample at age 2 years. The 16S rRNA gene sequencing was completed as described in the work of Huda et al. (9), and results were processed using the QIIME2 DADA2 pipeline (51, 52). These results were used to select infants low in *Bifidobacterium* (<20% relative abundance) or high in *Bifidobacterium* (>65% relative abundance) in early life (age week 6, 11, or 15) for whole metagenomics sequencing. Only a single early-life sample per infant was included. The high prevalence of *Bifidobacterium* dominance of the gut microbiota in this cohort resulted in a limited number of low-*Bifidobacterium* samples available for inclusion. In addition, reliable information on whether infants received antimicrobial agents was unavailable and so could not be included in analysis.

### DNA extraction and whole-metagenomics sequencing.

DNA was extracted as described in the work of Huda et al. (9) and sent to the UC Berkeley Functional Genomics Laboratory for library preparation and subsequent Illumina sequencing at the UC Berkeley Genomics Sequencing Laboratory. Prior to sequencing, each sample was sheared using the 150-bp setting of the Diagenode Bioruptor and then purified and concentrated with the Qiagen MinElute cleanup kit. End repair, A tailing of DNA fragments, and adapter ligation were performed using the Kapa Hyper Prep library kit. Next, 9 cycles of indexing PCR were performed using the Kapa Hi-Fi HotStart amplification kit. Cleanup and dual-solid phase reversible immobilization (SPRI) size selection were completed using AMPure beads. Libraries were checked for quality on the AATI fragment analyzer (Advanced Analytical Technologies, Inc.), quantified with Kapa Illumina Library quantitative PCR on a Bio-Rad CFX Connect, and pooled in equimolar amounts. Sequencing was completed at 3 nM using the Illumina HiSeq4000 with 150-bp paired-end reads. Bcl files were converted to demultiplexed FastQ file format using the Illumina bcl2fastq v2.18 software.

Samples were sequenced in three separate sequencing runs. The first two runs consisted of a mix of high- and low-*Bifidobacterium* samples from early life, while the third run contained the samples from year 2.

### Analysis of antimicrobial resistance genes.

Organisms may have intrinsic, adaptive, or acquired AMR (53). Intrinsic resistance refers to inherent characteristics of the organism that result in resistance to an antimicrobial agent: for example, the outer membrane of Gram-negative organisms prevents agents from reaching their targets (53). Importantly, intrinsic resistance is not usually horizontally transferable (54). Adaptive resistance is when an organism can rapidly turn on a gene in response to environmental cues (53). Unlike intrinsic or acquired resistance, adaptive resistance will usually revert when the environmental trigger is removed (55). Acquired resistance is when a previously sensitive organism gains resistance to an antimicrobial agent either through new mutations or through acquisition of ARGs from other organisms (53). Acquired resistance is particularly problematic, as part of the problem with carriage of AMR organisms is that ARGs can transfer between both closely and distantly related taxa (56), and many of the resistance genes found in commensal human gut flora are identical at the nucleotide level to resistance genes in human pathogens (57). The differences in importance between intrinsic and acquired AMR have led to the creation of databases with different focuses. For example, the ResFinder database focuses exclusively on acquired resistance genes (58), while the MEGARes database includes both intrinsic and acquired AMR (36). Despite this ubiquity of ARGs in bacteria, not all bacteria carry ARGs in general, or acquired ARGs specifically, at comparable levels.

Prior to analyzing reads with ResFinder or MEGARes, host subtraction was completed on each sample using BMTagger in BMTools v. 1 (59) and the GRCh38 build of the human genome. Reads were trimmed using Trimmomatic v. 0.36 with a sliding window of 4 bp, a minimum average quality of 15, and a minimum length of 99 bp. Paired-end reads were then merged using FLASH v 1.2.11 (60). The trimmed reads where both ends survived trimming were then assembled using MEGAHIT v. 1.0.6 (61), and the resulting final contigs were uploaded to ResFinder 3.0 (58) to identify the presence or absence of acquired ARGs. Contigs were not filtered by length because ARGs may be found on plasmids and plasmids may be as small as 1 kb (62). The numbers of unique ARGs present in high- and low-*Bifidobacterium* infants in early life were compared in R 3.4.3 statistical software (63) using Kruskal-Wallis tests for both early-life and year 2 samples. The number of unique ARGs present in the early-life high- and low-*Bifidobacterium* samples was then divided by the number of reads in the sample to account for the differences in read depth observed in the early-life samples, and numbers were compared using Kruskal-Wallis tests. To test if differences in ARGs in early life may be related to differences in delivery mode, a stratified analysis was completed comparing ARG counts by delivery mode in high- and low-*Bifidobacterium* infants. The assembled contigs identified as containing an ARG by ResFinder were compared with the NCBI nucleotide collection (nr/nt) (33) using a custom BLAST script (https://github.com/akre96/ResistBlast) to identify the most probable taxon of origin for each ARG. For each ARG, the contig containing the ARG was run through NCBI BLAST, and the top three species hits with at least 90% identity match were returned. As ResFinder is focused on transferable ARGs which can be shared across taxa, the family of each of the top three hits was identified. If all three hits were from the same family, the ARG was considered to originate in that family. If the top three hits originated from two or more families, the origin or the ARG was considered mixed. If ResistBlast returned no hits or if all hits were to uncultured bacterium, the family of origin was considered to be unknown. The percentage of ARGs in each sample mapping to each origin was calculated by dividing the number of ARGs mapping to a particular origin in each sample, dividing by the total number of ARGs in that sample, and multiplying by 100.

Next, the FLASH merged reads were analyzed using AMR++ (resistomeanalyzer v. 1) and the MEGARes v 1.01 database using default settings (36) to determine the total number of reads mapping to ARGs. To normalize the number of AMR reads, the merged reads were analyzed using METAXA2 v. 2.1.3 (64) to count the number of reads mapping to bacterial 16S rRNA genes. The normalized abundance of ARGs was then calculated for each sample as described in the work of Li et al. (35) using the formula

$$\text{Abundance}=\overset{n}{\underset{1}{\sum }}\frac{N\left(\text{AMR}\right)\times L\left(\text{reads}\right)/L(\text{AMRref})}{N\left(16\text{S}\right)\times L(\text{reads})/1,432}$$

where *n* is each individual ARG in the MEGARes database, *N*(AMR) is the number of reads mapping to ARGs according to AMR++, *L*(reads) is the length of the sequencing reads and cancels out of the equation because the length is the same for both the AMR reads and the 16S reads, *L*(AMRref) is the length of the AMR reference gene in the MEGARes database, *N*(16S) is the number of reads mapping to 16S rRNA gene bacterial reads by METAXA2, and 1,432 is the average length of a 16S rRNA gene sequence in the Greengenes database. Differences in total AMR abundance between high- and low-*Bifidobacterium* infants in early life were tested in both early-life samples using the Kruskal-Wallis test in R (63). An advantage of the MEGARes database is its acyclic classification of ARGs into groups, mechanisms, and classes. We were then able to test for differences between high- and low-*Bifidobacterium* infants in ARG abundance at the gene, group, mechanism, and class levels of the MEGARes database using a Bonferroni correction for multiple comparisons at each level and Kruskal-Wallis tests. As with the ResFinder analysis, a stratified analysis (stratified by *Bifidobacterium* level) was used to test for an association between AMR levels and delivery mode in early life. To confirm the results of the early-life Bangladeshi comparison, FLASH merged reads were also processed using ShortBRED (37) using default settings and the 2017 CARD marker gene database prebuilt by the authors.

Sequencing results from year 2 samples were processed in the same manner as early-life samples for the ResFinder analysis. For the abundance analysis, only the classes, mechanisms, groups, and genes significantly different in early-life samples were compared in later-life samples to determine if any differences in AMR in early life are maintained at later time points. Early-life and year 2 total abundances of AMR were then compared using a Wilcoxon paired test.

### Total bacteria, total *Bifidobacterium*, and total *Enterobacteriaceae* quantification.

Quantitative PCR (qPCR) was conducted to determine the absolute abundance of total bacteria and total *Bifidobacterium*. Briefly, DNA extracted from stool was diluted 1:10 before use. The standard curves for qPCR were generated using a 10-fold dilution series of DNA extracted from Escherichia coli sldp 38-1 (total bacteria) or Bifidobacterium longum subsp. *infanti*s ATCC 15697 (total *Bifidobacterium*) in liquid cultures at late exponential phase for which cell numbers were determined by quantitative culture. Total bacterial qPCR was completed using a melting temperature (*T_m_*) of 60°C using the 27F (5′AGAGTTTGATCCTGGCTCAG3′) and 355R (5′CTGCTGCCTCCCGTAGGAGT3′) primers described by Cariveau et al. (65). Total *Bifidobacterium* qPCR was completed using a *T_m_* of 58°C using the genus *Bifidobacterium* F (5′TCGCGTC[C/T]GGTGTGAAAG3′) and genus *Bifidobacterium* R (5′CCACATCCAGC[A/G]TCCAC3′) primers described by Krumbeck et al. (66). Total *Enterobacteriaceae* qPCR was completed using a *T_m_* of 56°C using the family *Enterobacteriaceae* F (5′CATTGACGTTACCCGCAGAAGAAGC3′) and family *Enterobacteriaceae* R (5′CTCTACGAGACTCAAGCTTGC3′) primers as described by Oh et al. (67). Total abundances of bacteria, total abundances of *Bifidobacterium*, and total abundances of *Enterobacteriaceae* were compared between the high- and low-*Bifidobacterium* groups using a Kruskal-Wallis test. The correlation between early-life bacterial abundance (total, *Bifidobacterium*, and *Enterobacteriaceae*) and normalized total AMR abundance (from AMR++) was calculated using Spearman’s test. Early-life and year 2 total bacteria and total *Bifidobacterium* levels were stratified by *Bifidobacterium* level, and only infants with both an early-life and a year 2 sample were included for statistical testing using a Wilcoxon paired test.

### Swedish cohort.

To confirm findings in the Bangladeshi cohort in an unrelated cohort, samples from the study by Bäckhed et al. (30) of the human gut microbiome in Swedish infants during the first year of life were downloaded from EBI’s Sequence Read Archive under the accession code ERP005989. Briefly, this cohort enrolled 100 mother-infant dyads and used WGS to examine the development of the infant microbiome from birth until age 12 months. For this study, the infant month 4 and month 12 samples were included. After downloading the month 4 and 12 sequencing files, data were processed using AMR++, which includes a normalization step to adjust for differences in read depth, with the same criteria as the Bangladeshi cohort except that the FLASH merge step was skipped due to a longer insert size. As samples were sequenced without selection based on *Bifidobacterium* levels, correlation between read depth and *Bifidobacterium* relative abundance was tested using Spearman’s correlation test at both months 4 and 12. Then, regression using the log-transformed total AMR was completed in R 3.4.3 using the glm command and including the number of times that infants that were exposed to antibiotics before age 4 months and the relative abundance of *Bifidobacterium* in the month 4 samples as predictor variables. Then, to more directly compare results of the two cohorts, samples were classified as high or low *Bifidobacterium* using the METAXA2 results on the month 4 samples using the same criteria as for the 16S rRNA gene sequencing on the Bangladeshi cohort. The abundances of AMR in the month 4 and month 12 samples were calculated using the AMR++ pipeline as described above.

### Accession number(s).

Host subtracted data are available on the NCBI SRA under accession number SRP133760.

10.1128/mSphere.00441-18.10 TABLE S5 Median normalized abundance for AMRs identified by MEGARes that were significantly different between the high- and low-*Bifidobacterium* samples in Sweden. *P* value for significant difference at the class level was 0.00294, at the mechanism level was 0.00106, and at the group level was 0.000289. Download Table S5, PDF file, 0.1 MB. Copyright © 2018 Taft et al. 2018 Taft et al. This content is distributed under the terms of the Creative Commons Attribution 4.0 International license.
