# Supplementary figures and images for: Bifidobacterial Dominance of the Gut in Early Life and Acquisition of Antimicrobial Resistance
Source: mSphere. 2018 Sep 26;3(5):e00441-18. doi: 10.1128/mSphere.00441-18 (PMC6158511; doi:10.1128/mSphere.00441-18)

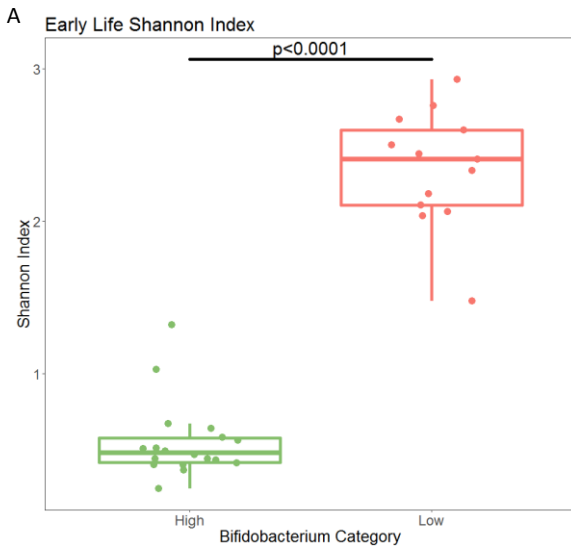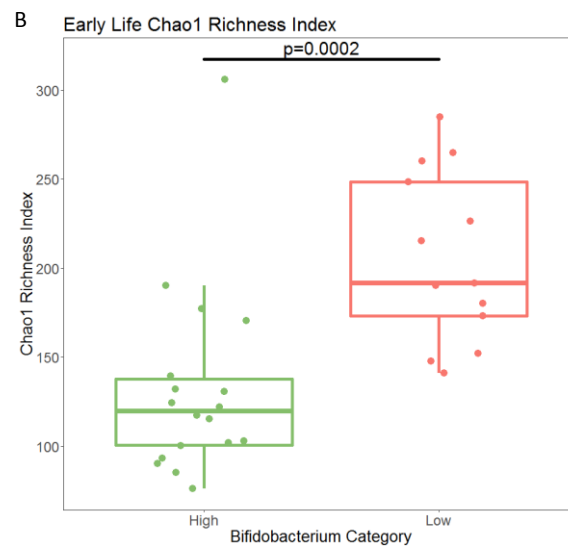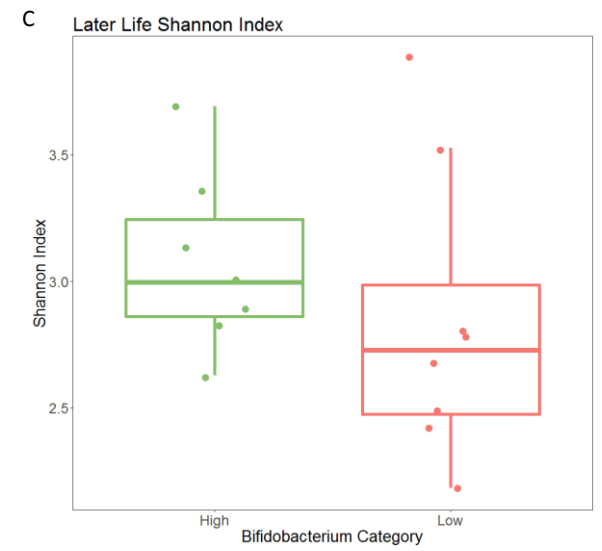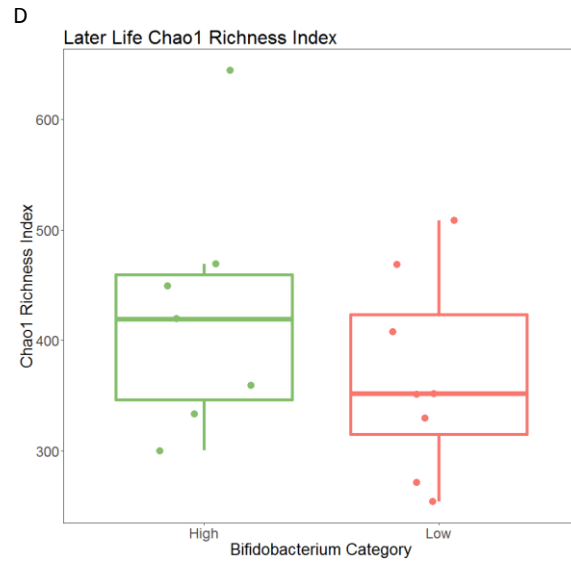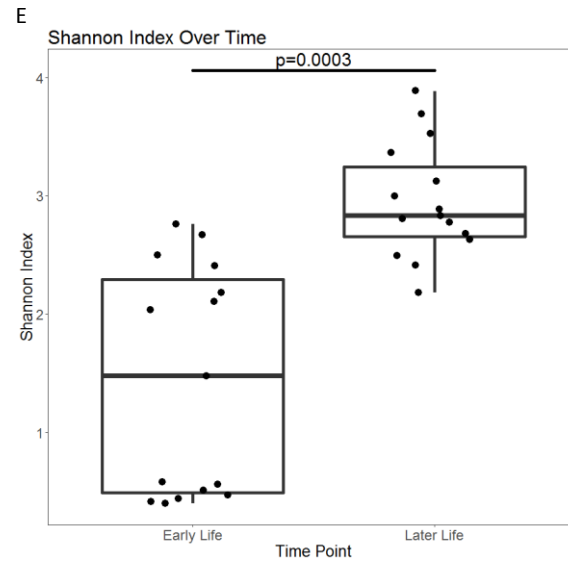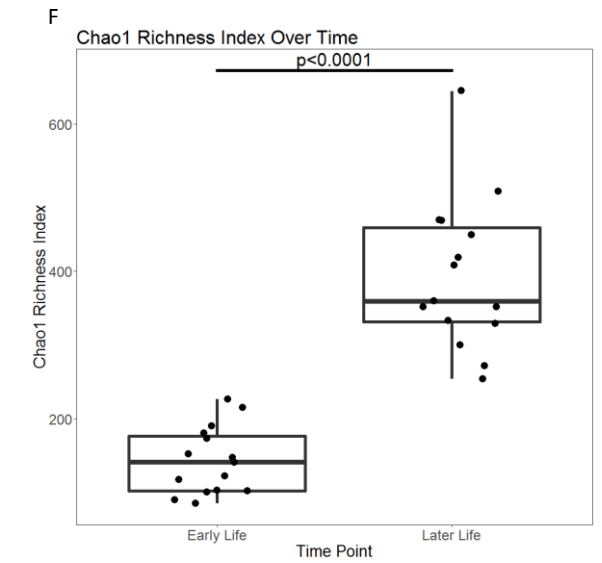

Supplement: FIG S1 [file sph005182646sf1.pdf]

# ResFinder Number of ARGs Normalized by Read Depth

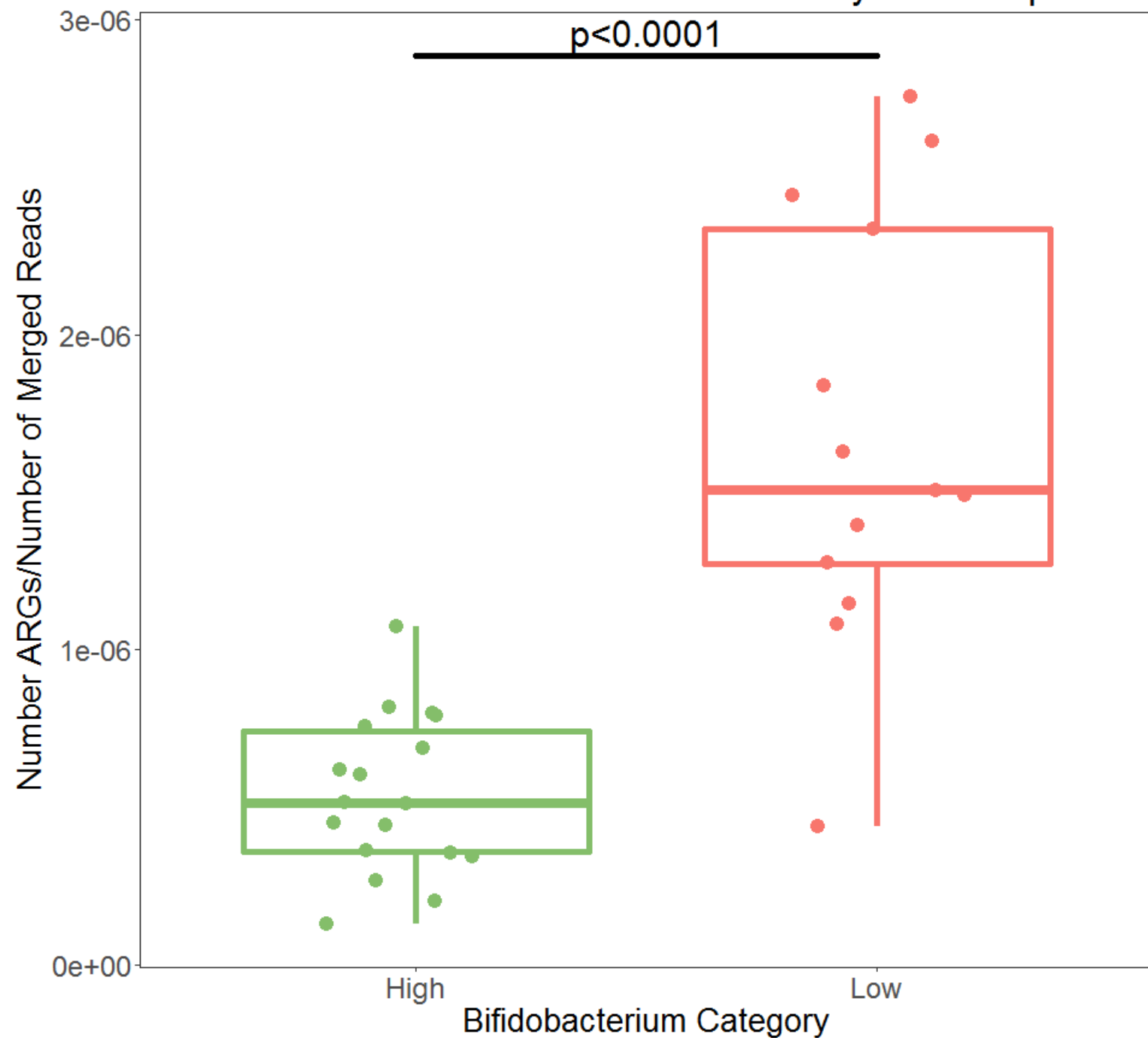

Supplement: FIG S3 [file sph005182646sf3.pdf]

A

## Total Bacteria Levels

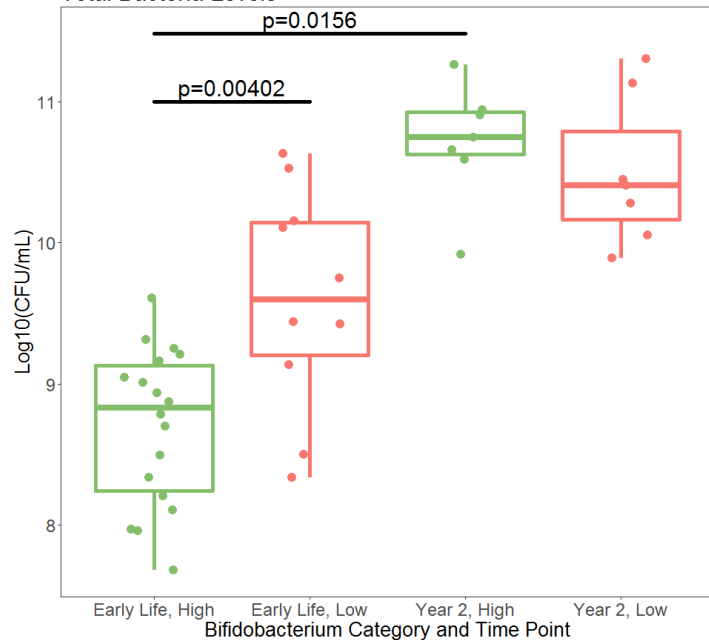

B

## Bifidobacterium Levels

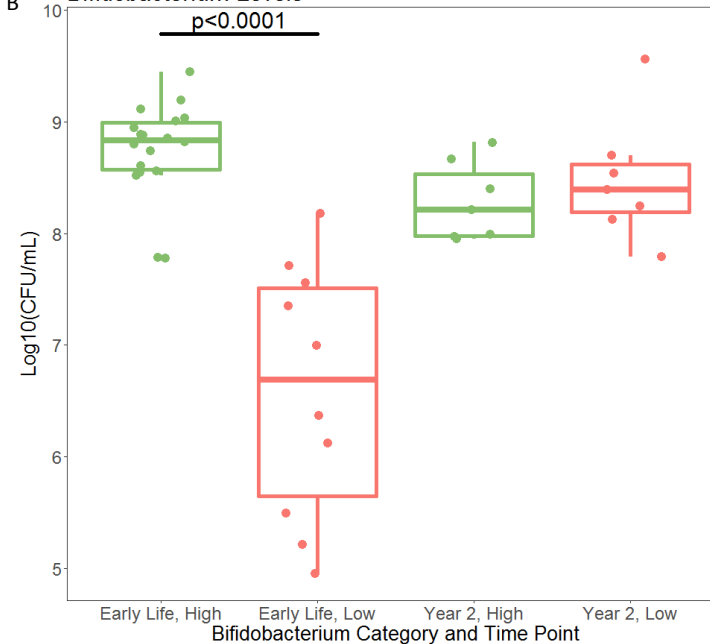

C

## Enterobacteriaceae Levels

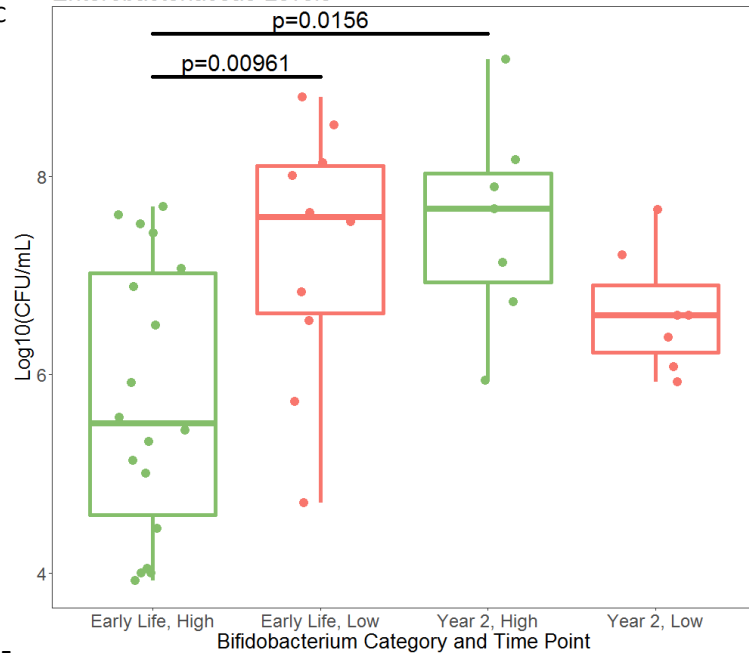

Supplement: FIG S5 [file sph005182646sf5.pdf]
