# Supplementary material for: Bifidobacterial Dominance of the Gut in Early Life and Acquisition of Antimicrobial Resistance
Source: mSphere. 2018 Sep 26;3(5):e00441-18. doi: 10.1128/mSphere.00441-18 (PMC6158511; doi:10.1128/mSphere.00441-18)
Supplement: FIG S2 [file sph005182646sf2.pdf]

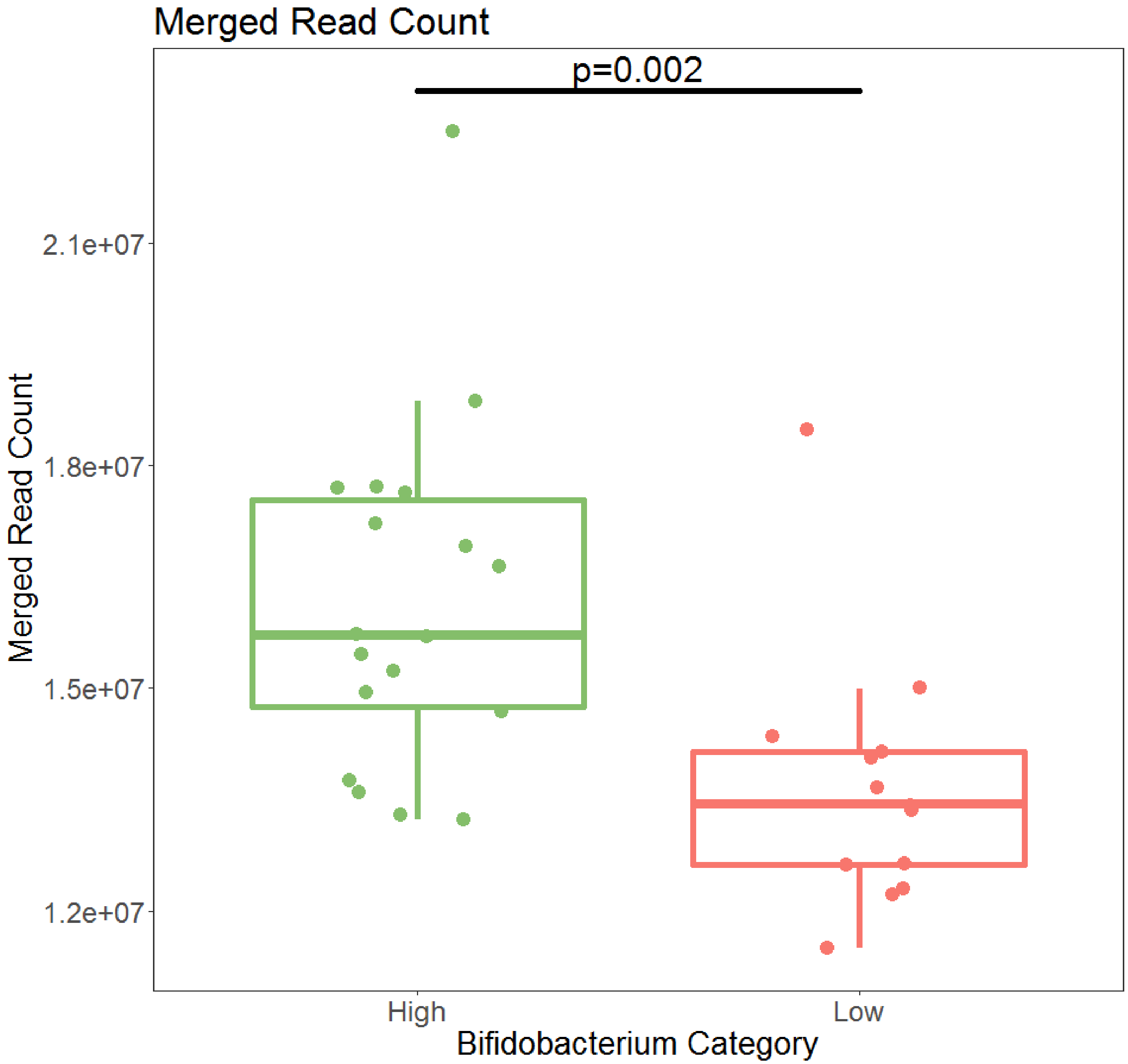
Merged Read Count
p=0.002
2.1e+07
1.8e+07
1.5e+07
1.2e+07
Merged Read Count
High
Low
Bifidobacterium Category

SF2
