# Supplementary material for: Bifidobacterial Dominance of the Gut in Early Life and Acquisition of Antimicrobial Resistance
Source: mSphere. 2018 Sep 26;3(5):e00441-18. doi: 10.1128/mSphere.00441-18 (PMC6158511; doi:10.1128/mSphere.00441-18)
Supplement: TABLE S1 [file sph005182646st1.pdf]

Sample Name	Run	Raw Reads	Non-Human Reads	Trimmed Reads	Merged Reads	Number of 16S Reads by METAXA2	Number of ARGS by AMR++	Number of Contigs	Total Length (bp)	N50 (bp)	Median Fold Coverage
1064w11	1	14380221	14334743	13315456	12636757	27217	125267	50127	61051555	2288	2.6
1053w11	1	19629451	13661611	12710795	12219836	41588	83246	18082	29170214	588	3.4
1063w11	1	17378110	17362608	16081929	14995458	51707	66697	47426	60953333	2162	2.6
1076w11	1	15183617	15028195	14096572	13662359	35623	54376	35061	34487584	1861	2.1
2055w11	1	20591207	20289114	18959385	17691644	45367	20715	16566	20554102	1686	2.9
2056w11	1	17851881	17842489	16753754	15467766	39764	20528	3649	5874235	110	3.1
2058w11	1	17536887	17075871	15348380	13232359	30079	22278	9453	20405925	127	3.3
1068w11	1	22948608	22937547	21340052	17711835	39130	17781	12990	16190699	1585	2.4
2053w11	1	17300496	17279551	16216059	15230690	38536	30236	21226	23308243	940	2.4
1065w15	1	17084988	17060815	16132190	15703015	33157	22476	24657	15976762	5869	3.8
1073w11	1	19539299	19523131	17845554	16912413	42543	7896	6690	6467660	702	3.1
2057w11	1	23762708	23735806	21917539	18864612	41789	29713	6878	15681795	205	2.8
3003w15	1	19611190	19583188	18284763	17640081	41146	10401	20612	14146450	3934	2.3
3015w15	1	24580377	24539514	23196783	22504680	42133	66358	15988	16213571	1061	2.3
3016w15	1	25128737	21614107	19722233	18472920	46586	158941	35815	66449697	1449	4.4
3112w6	2	13672450	13664712	12765848	12301455	28206	11725	35487	58153682	1457	3.1
3047w6	2	24580990	24536103	21607252	16643043	44571	32986	7221	15639390	95	2.4
3113w6	2	14904714	14836311	13968692	13355094	28575	174261	26901	33982241	606	3.4
3084w6	2	18577575	18568806	16885262	14693982	33654	10113	16156	21241431	1217	3.6
3032w6	2	20735595	20686340	17749861	14342661	28429	43880	25420	43572967	422	2.6
3060w6	2	17859309	17845300	15896252	13750615	35381	56254	11625	15900095	566	2.8
1089w6	2	16390946	16380210	14971412	11494270	18592	5808	20360	62273104	292	2.9
3073w6	2	19683953	19636243	17876558	13600319	33139	15030	7344	10708874	106	2.4
1055w6	2	15120882	15077876	13950310	13292318	23214	6089	6735	12770225	216	3.2
3034w6	2	21366435	20788378	18196493	13428885	46111	66789	39406	69214072	1116	2.8
1026w6	2	20953772	20918779	18574136	14147259	33159	287176	22061	34698627	1685	3.5
1108w6	2	19746073	18583770	16849105	12615808	46164	60155	32133	55534922	1470	3.0

2085w6	2	17747630	17466203	15966043	14070444	55386	14429	35964	51631713	1961	3.6
1078w6	2	20863345	20845831	18501901	14943498	37023	11505	9418	7550175	948	2.2
1067w6	2	25835804	25715076	22993270	17211115	40940	17625	5935	12125208	263	4.4
2003w6	2	22693868	22592569	19877432	15721450	42391	21396	15715	18037980	1246	2.7
3112Y2	3	15915495	15894931	15223603	15079932	39667	8482	111564	103013982	10894	2.7
3047Y2	3	18140211	18117511	17234928	17010066	41833	85055	194976	195010593	15389	2.8
3113Y2	3	20676570	20652394	19697279	19315250	47016	19077	90188	89601018	7942	2.7
3084Y2	3	23071393	23054841	21776412	20414487	53136	17249	129155	134064923	10109	2.9
3032Y2	3	16955520	16925521	15990438	15596045	53981	36658	157385	182063022	11466	3.0
3060Y2	3	17473920	16271611	15486962	15024539	50609	35666	119015	130634566	9711	3.1
1089Y2	3	16735419	16724259	15781718	15074732	45944	13276	222477	230296407	22196	3.0
3073Y2	3	18772114	18754532	17938554	17544385	47704	31636	144553	172559452	10106	2.9
1055Y2	3	17137212	17124183	16114914	15740123	51409	26164	231719	221387099	25152	2.8
3034Y2	3	18472422	18463327	17729658	17441432	42699	18544	75729	75170747	6029	2.7
1026Y2	3	16271657	16262006	15619917	15387886	45703	8510	45972	78724028	1090	2.6
1108Y2	3	18770895	16968867	16255801	16029212	52904	40721	106673	109260311	9722	3.0
2085Y2	3	17103018	16605614	15854678	15448880	63791	14623	82184	96467510	5770	3.1
1078Y2	3	13743837	13736308	13066373	12641209	37407	9706	72437	83115888	5148	2.8
1067Y2	3	15417592	15400700	14669310	13838867	34455	38958	123940	138911813	10522	2.7

Supplemental Table S1
