# Supplementary material for: Bifidobacterial Dominance of the Gut in Early Life and Acquisition of Antimicrobial Resistance
Source: mSphere. 2018 Sep 26;3(5):e00441-18. doi: 10.1128/mSphere.00441-18 (PMC6158511; doi:10.1128/mSphere.00441-18)
Supplement: TABLE S2 [file sph005182646st2.pdf]

Resistance Class	High *Bifidobacterium* samples n=18	Low *Bifidobacterium* samples n=13
Aminoglycoside	9	11
Beta-lactam	10	12
Fluoroquinolone	4	10
Fosfomycin	3	6
Glycopeptide	0	2
MLS	18	13
Nitroimidazole	0	1
Phenicol	5	5
Sulphonamide	6	11
Tetracycline	16	13
Trimethoprim	7	9

Supplemental Table S2
