# Supplementary material for: Bifidobacterial Dominance of the Gut in Early Life and Acquisition of Antimicrobial Resistance
Source: mSphere. 2018 Sep 26;3(5):e00441-18. doi: 10.1128/mSphere.00441-18 (PMC6158511; doi:10.1128/mSphere.00441-18)
Supplement: TABLE S3 [file sph005182646st3.pdf]

Class	Mechanism	Group	Gene
Aminocoumarins	Aminocoumarin-resistant DNA topoisomerases	PARE	None
Aminoglycosides	Aminoglycoside efflux pumps	ACRD	None
	Aminoglycoside efflux regulator	None	None
Bacitracin	Undecaprenyl pyrophosphate phosphotase	BACA	None
Betalactams	Class A betalactamases	CTX	1268|AB976602.1|AB976602|betalactams|Class_A_betalactamases|CTX
	Penicillin binding protein	AMPH	None
Cationic antimicrobial peptides	None	None	None
Elfamycins	EF-Tu inhibition	TUFAB	CARD|pvgb|AE014075|3901532-3902762|ARO:3003438|Escherichia|Elfamycins|EF-Tu_inhibition|TUFAB|RequiresSNPConfirmation
			CARD|pvgb|CP000647|4764664-4765849|ARO:3001312|elfamycin|Elfamycins|EF-Tu_inhibition|TUFAB|RequiresSNPConfirmation
Fluoroquinolones	Fluoroquinolone-resistant DNA topoisomerases	PARC	None
		GYRB	None
		GYRA	None
MLS	23S rRNA methyltransferases	ERMX	CARD|phgb|AF024666|2207-2969|ARO:3000596|ErmX|MLS|23S_rRNA_methyltransferases|ERMX
			MLS|ermX_3_U21300|MLS|23S_rRNA_methyltransferases|ERMX
			MLS|ermX_2_X51472|MLS|23S_rRNA_methyltransferases|ERMX
			MLS|ErmX|M36726|296-1150|855|MLS|23S_rRNA_methyltransferases|ERMX
Multi-drug resistance	MDR mutant porin proteins	OMPF	None
	MDR regulator	MARR	None
		HNS	CARD|phgb|NC_002695|1737553-1737967|ARO:3000676|H-NS|Multi-

			drug_resistance|MDR_regulator|HNS
		EVGA	None
		CRP	CARD|phgb|AP009048|4153663-4154296|ARO:3000518|CRP|Multi-drug_resistance|MDR_regulator|CRP
		CPXAR	None
		CPXA	None
		BAER	None
		ASMA	None
	Multi-drug efflux pumps	ACRB	11|DQ679966.1|DQ679966|Multi-drug_resistance|Multi-drug_efflux_pumps|ACRB
		MDTB	None
		EMRR	None
		EMRK	None
		EMRD	None
		EMRB	None
Rifampin	Rifampin-resistant beta-subunit of RNA polymerase RpoB	RPOB	None
Sulfonamides	Sulfonamide-resistant dihydropteroate synthases	FOLP	None

Supplemental Table S3
