# Supplementary material for: Bifidobacterial Dominance of the Gut in Early Life and Acquisition of Antimicrobial Resistance
Source: mSphere. 2018 Sep 26;3(5):e00441-18. doi: 10.1128/mSphere.00441-18 (PMC6158511; doi:10.1128/mSphere.00441-18)
Supplement: FIG S4 [file sph005182646sf4.pdf]

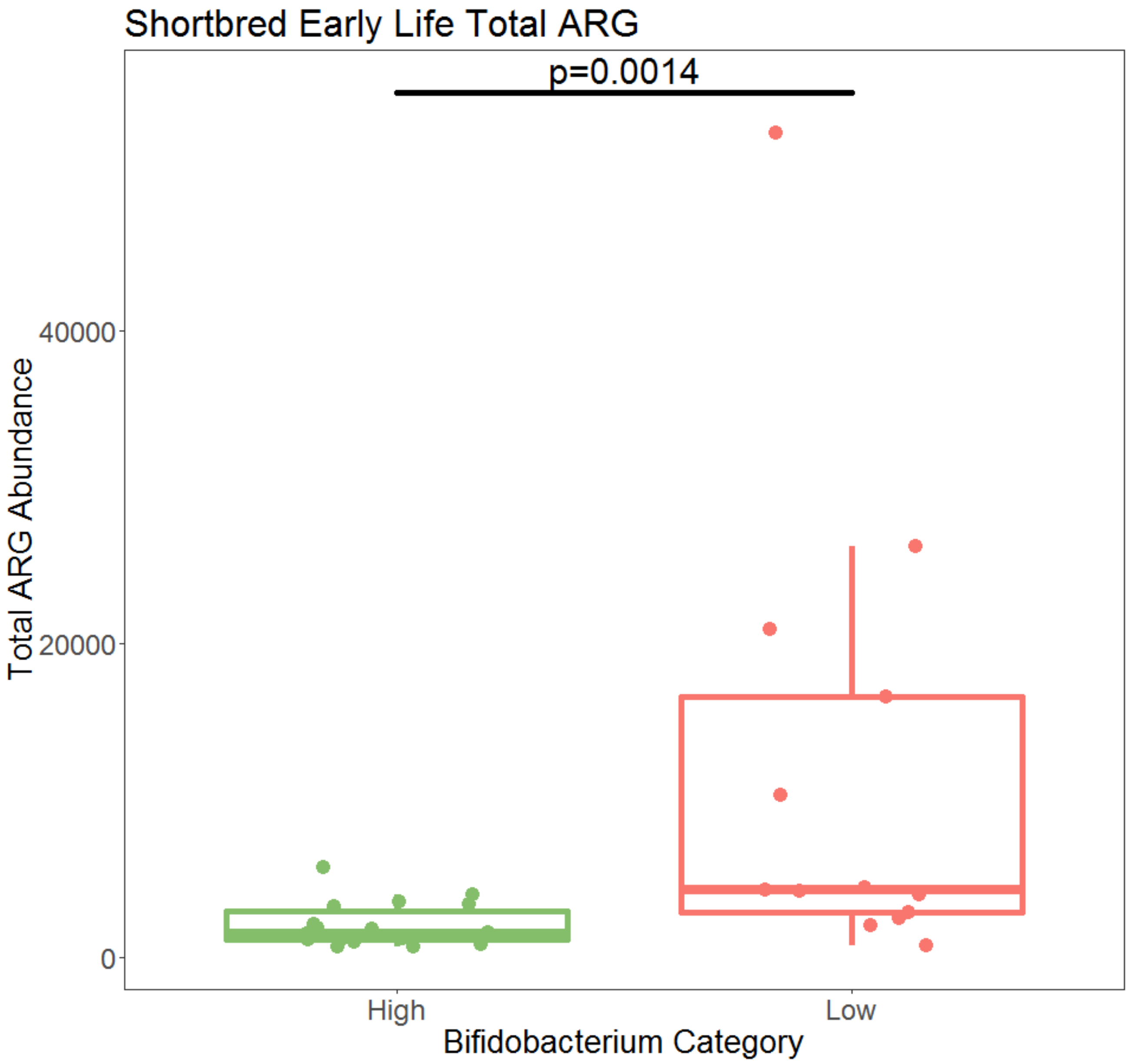

Shortbred Early Life Total ARG
p=0.0014
Total ARG Abundance
40000
20000
0
High
Low
Bifidobacterium Category

SF4
