# Supplementary material for: Bifidobacterial Dominance of the Gut in Early Life and Acquisition of Antimicrobial Resistance
Source: mSphere. 2018 Sep 26;3(5):e00441-18. doi: 10.1128/mSphere.00441-18 (PMC6158511; doi:10.1128/mSphere.00441-18)
Supplement: TABLE S4 [file sph005182646st4.pdf]

Subject Number	Run Accession	Month	Raw Reads	Non-Human Reads	Trimmed Reads	Number of 16S Reads by METAXA2	Number of ARGS by AMR++
1	ERR525721	4	25194766	25194552	12284258	16318	31746
7	ERR526069	4	14665521	14665409	4977246	11466	191452
9	ERR526085	4	24636188	24636016	12284893	21081	367866
10	ERR525693	4	21754446	21754303	12746631	20233	87199
12	ERR525702	4	17001574	16952581	10338601	17225	173217
18	ERR525750	4	18834353	18834258	7024546	13249	58704
26	ERR525805	4	16814591	16814408	4189035	11803	59742
38	ERR525885	4	18764372	18763798	8296093	14406	137641
39	ERR525897	4	20399449	20398301	8642293	14126	209450
42	ERR525905	4	20194674	20194259	10242776	8115	75616
45	ERR525909	4	22106860	22106766	14622027	17595	54207
51	ERR525929	4	22462043	22461830	14022419	17994	41987
53	ERR525949	4	24742763	24742484	15056810	22842	308837
70	ERR526065	4	22383268	22383147	10490163	15785	11057
78	ERR526071	4	26377908	26377742	12932816	16593	122841
82	ERR526077	4	17592983	17592362	11295251	14278	21032
87	ERR526081	4	29211695	29211122	18303370	31011	237214
103	ERR525690	4	16231842	16230748	7207270	15467	134221
105	ERR525695	4	17722853	17719545	6744742	14664	139598
128	ERR525704	4	19819050	19815942	11091745	21103	247666
130	ERR525710	4	23491541	23491367	11362899	18640	251207
133	ERR525714	4	18694275	18693885	9435111	16963	63842
137	ERR525718	4	20649572	20649468	9538590	10949	21838
150	ERR525723	4	23589204	23588970	15184081	21618	35737
157	ERR525727	4	24786867	24783121	15834911	25994	55010
172	ERR525731	4	20565027	20563964	13904636	23956	67054
179	ERR525735	4	24354870	24354505	15227890	18334	26179
180	ERR525739	4	21734489	21734306	13699461	21507	83768

181	ERR525744	4	18075345	18074795	6946877	12770	77558
184	ERR525748	4	15554285	15554204	4195449	5678	1499
195	ERR525755	4	16983205	16983132	6263191	7947	18274
201	ERR525761	4	15747516	15744159	5004689	10920	95565
203	ERR525765	4	13432721	13432235	3832479	8203	14330
224	ERR525769	4	18007748	18007676	8551124	9339	49572
229	ERR525773	4	16364265	16355319	4296653	7820	152211
238	ERR525777	4	13481397	13472907	4613277	10398	7839
240	ERR525781	4	13584665	13584303	5241981	6122	97153
241	ERR525785	4	20217874	20216782	7036977	8552	16134
244	ERR525789	4	15424240	15423978	3884723	5216	10403
256	ERR525793	4	17656215	17636264	3609736	9898	137870
258	ERR525797	4	20378830	20378661	8399971	10162	280996
263	ERR525802	4	21101296	21101217	7684060	5955	23441
265	ERR525807	4	19356333	19354945	3165935	6168	129766
268	ERR525811	4	25236501	25235234	16845025	26285	92579
272	ERR525817	4	28617163	28278056	21133001	25257	32332
274	ERR525821	4	22950635	22950538	10795482	9329	121757
275	ERR525825	4	14749523	14748277	3915596	11161	50209
276	ERR525829	4	18894693	18893873	9214328	16380	110532
281	ERR525833	4	20793881	20793458	12325290	26301	35660
282	ERR525837	4	18562146	18560671	8174618	18686	29345
326	ERR525841	4	40102129	40100769	17665878	37265	789539
332	ERR525845	4	22987886	22987556	13784211	13610	25399
335	ERR525849	4	18721524	18721154	10216750	22285	19169
338	ERR525853	4	14973436	14964017	6995474	13761	43250
341	ERR525857	4	17175377	17175273	10304395	15986	34311
343	ERR525861	4	23176026	23175801	14771318	16599	51387
345	ERR525865	4	20695891	20695568	9987230	20741	3666
350	ERR525869	4	21510512	21509385	13738856	21935	71875

367	ERR525873	4	21016509	21014215	8474472	17804	30408
377	ERR525877	4	20603169	20602955	11498568	22920	439031
383	ERR525882	4	20920669	20920324	11567310	11931	169178
385	ERR525887	4	15540860	15514827	3893391	10031	22580
387	ERR525891	4	19208410	19206184	6620312	16435	214669
397	ERR525899	4	21836384	21835316	9571950	19529	193381
503	ERR525913	4	21725058	21723105	12567917	22739	229100
504	ERR525917	4	18795997	18795444	11743009	21956	220361
507	ERR525921	4	22694224	22693855	13558992	25945	56900
511	ERR525926	4	25827701	25827125	17423051	22198	14207
521	ERR525933	4	24052264	24051991	16104394	19280	52700
526	ERR525937	4	19814600	19814519	10394341	19179	10480
527	ERR525941	4	22132147	22130183	11638240	17291	82630
532	ERR525946	4	28205978	28205424	19747614	38405	62268
536	ERR525951	4	19182588	19181722	7321519	13830	141796
544	ERR525957	4	24580719	24571411	13407706	19905	51131
546	ERR525961	4	23901614	23901023	11682278	14118	141195
549	ERR525965	4	21654153	21652456	11891021	17750	452589
560	ERR525969	4	16904970	16901177	5336046	7893	155531
565	ERR525973	4	20317065	20317011	9842102	10589	7076
566	ERR525977	4	16829140	16828464	5481597	14185	132358
567	ERR525981	4	16291823	16291019	6137067	10264	114007
570	ERR525985	4	22431839	22431691	11858854	10240	8199
572	ERR525989	4	18505696	18494607	8306002	9273	186716
577	ERR525993	4	24807919	24807822	14577389	7086	23915
582	ERR525997	4	20079441	20079370	11167056	16982	15837
585	ERR526001	4	15888603	15888573	4666937	10587	462
587	ERR526005	4	24951100	24948246	15713391	21154	40845
589	ERR526009	4	24511117	24509536	16542846	14167	11532
590	ERR526013	4	21984137	21983641	13355506	17927	9531

591	ERR526017	4	19243196	19243117	9933453	11562	13078
598	ERR526021	4	19352743	19352378	10333686	11200	80951
599	ERR526025	4	4851194	4252552	1101234	3213	2212
608	ERR526029	4	17555367	17554837	8934027	22286	3985
615	ERR526033	4	14404982	14404823	6689290	13875	46563
622	ERR526037	4	12941365	12941152	5067706	14251	11048
624	ERR526041	4	17966980	17963481	13362894	25511	132413
626	ERR526045	4	16671736	16671594	9947420	20002	42328
633	ERR526049	4	17434745	17423546	5081769	8573	91581
635	ERR526053	4	19007307	19006645	8498496	26810	76377
637	ERR526057	4	20970481	20970290	11112983	21201	39980
640	ERR526061	4	18784037	18783374	9165379	13128	22210
1	ERR525700	12	28321904	28321728	18218009	17514	38203
7	ERR526068	12	18026372	18026156	11052593	16928	11435
9	ERR526084	12	25993558	25991010	15338747	20453	172137
10	ERR525688	12	22028790	22028621	13203373	19980	9114
12	ERR525701	12	17326678	17326540	11021694	13042	14438
18	ERR525743	12	17188228	17187662	10244666	12217	14241
26	ERR525800	12	22809092	22808684	13087690	18548	8489
38	ERR525880	12	20725008	20724796	10947454	17965	23639
39	ERR525896	12	25375033	25374783	13552887	17832	14298
42	ERR525904	12	24638741	24638600	11901578	16307	19938
45	ERR525908	12	17609422	17609250	9233467	14709	20443
51	ERR525925	12	23497887	23497682	13448728	20057	19040
53	ERR525944	12	23640240	23639895	13704685	22122	98940
70	ERR526064	12	27541520	27541007	16469725	24603	28552
78	ERR526070	12	25595188	25594607	14531720	19826	12543
82	ERR526076	12	21230391	21229971	13593758	15925	3101
87	ERR526080	12	19199619	19198915	9349971	14654	13403
103	ERR525689	12	19454303	19453539	8798851	16463	13337

105	ERR525694	12	24033243	24025570	13856052	19175	21394
128	ERR525703	12	22596350	22595798	13266919	20064	13634
130	ERR525709	12	30170884	30170399	18763296	29164	158048
133	ERR525713	12	20682884	20682375	12204298	18045	16240
137	ERR525717	12	23732648	23731569	14951280	21992	91734
150	ERR525722	12	22891728	22891522	16246375	21953	13185
157	ERR525726	12	22820000	22819352	15460885	18161	14550
172	ERR525730	12	20208215	20207848	13172505	18547	15646
179	ERR525734	12	23712408	23712246	15519602	17850	14925
180	ERR525738	12	21184891	21184690	12729702	20002	32135
181	ERR525742	12	19986731	19986546	13754578	15609	14743
184	ERR525747	12	19874881	19874283	9671227	12847	9867
195	ERR525754	12	23403029	23401203	12958857	19941	25437
201	ERR525760	12	14478555	14478409	7282092	9151	14624
203	ERR525764	12	14151631	14151498	8112341	8278	15660
224	ERR525768	12	22649046	22648760	12398925	12237	34397
229	ERR525772	12	25836375	25834953	15382536	17702	21493
238	ERR525776	12	9659590	9659451	5369354	7175	8211
240	ERR525780	12	10767887	10767807	4313893	6167	9542
241	ERR525784	12	22062926	22062501	9124377	11326	18408
244	ERR525788	12	20056630	20055925	7943174	11186	21130
256	ERR525792	12	18500507	18500358	6523449	9135	18700
258	ERR525796	12	22459374	22458685	10246664	10637	4799
263	ERR525801	12	21928159	21927947	9592804	9280	13053
265	ERR525806	12	16056041	16055826	7066127	7135	22124
268	ERR525810	12	27189033	27188341	19529465	34015	10884
272	ERR525816	12	23217659	23216854	17181731	32932	8156
274	ERR525820	12	20713740	20713670	14876782	20555	7869
275	ERR525824	12	21090996	21090031	12373407	20477	5693
276	ERR525828	12	21894440	21894323	15770466	28317	6165

281	ERR525832	12	17696651	17695240	10289913	11385	24843
282	ERR525836	12	19696964	19696851	14331162	20029	15737
326	ERR525840	12	20104441	20104205	13000922	39937	42439
332	ERR525844	12	21258771	21258415	13029739	15442	18404
335	ERR525848	12	20586920	20586837	15192783	21973	23279
338	ERR525852	12	20024287	20024095	12874205	16237	11533
341	ERR525856	12	30093707	30093546	21128182	23566	6254
343	ERR525860	12	23892230	23892153	16664743	17328	18251
345	ERR525864	12	20243857	20243788	13372149	17642	17506
350	ERR525868	12	27443227	27442784	17586229	22814	14980
367	ERR525872	12	24416912	24416792	13019541	12337	23201
377	ERR525876	12	25805022	25804799	17056473	18111	23056
383	ERR525881	12	21288339	21288173	12587303	15270	11403
385	ERR525886	12	21239299	21236865	12856591	27164	12334
387	ERR525890	12	23822706	23822321	14691816	10669	11838
397	ERR525898	12	25931829	25931454	17850368	28106	25446
503	ERR525912	12	25916694	25916576	16636150	29615	6766
504	ERR525916	12	21198122	21197898	12462626	25344	32030
507	ERR525920	12	20418847	20372104	12482863	30012	35456
511	ERR525924	12	26358742	26358520	17963107	26128	11498
521	ERR525932	12	21346305	21346039	13716890	20156	254343
526	ERR525936	12	28566343	28565450	19389213	32991	33547
527	ERR525940	12	24571976	24571849	14429311	18994	20308
532	ERR525945	12	22050125	22049931	10292944	8069	13509
536	ERR525950	12	22672541	22672403	13890196	17734	6250
544	ERR525956	12	18833744	18833607	9466730	12392	12291
546	ERR525960	12	23563522	23560724	12455634	13653	60666
549	ERR525964	12	20848748	20848437	13051919	18068	19522
560	ERR525968	12	24380091	24379991	13171797	11779	33744
565	ERR525972	12	19159905	19159732	10683977	12029	10812

566	ERR525976	12	20384401	20383835	13100563	29087	13092
567	ERR525980	12	20117599	20117462	9486354	12048	32761
570	ERR525984	12	21123753	21123455	11580462	10913	6698
572	ERR525988	12	19971035	19969828	10733711	11647	14230
577	ERR525992	12	19747178	19745468	11465382	22928	31369
582	ERR525996	12	19018644	19018318	12773102	20609	10947
585	ERR526000	12	22984277	22983443	13910937	25643	13068
587	ERR526004	12	25346535	25344984	17226019	19025	24882
589	ERR526008	12	23108605	23105273	15614034	26488	14523
590	ERR526012	12	24829138	24828441	14549049	17246	13457
591	ERR526016	12	27298560	27297661	16053899	14308	16804
598	ERR526020	12	18638157	18638004	10353662	14717	10352
599	ERR526024	12	14341675	14339493	10731332	27252	26813
608	ERR526028	12	16474049	16473381	10351670	18310	82465
615	ERR526032	12	10993609	10993445	7741369	16438	7566
622	ERR526036	12	13531425	13531246	10168130	16240	2439
624	ERR526040	12	17967179	17966746	13641519	22453	30212
626	ERR526044	12	15557149	15556769	10864833	14031	3293
633	ERR526048	12	19638485	19634340	7328782	12874	31574
635	ERR526052	12	17853944	17853836	12933561	19303	8281
637	ERR526056	12	28625656	28625415	15146213	16867	67728
640	ERR526060	12	20330013	20329955	9708052	9346	20649

Supplemental Table S4
