# Supplementary material for: Bifidobacterial Dominance of the Gut in Early Life and Acquisition of Antimicrobial Resistance
Source: mSphere. 2018 Sep 26;3(5):e00441-18. doi: 10.1128/mSphere.00441-18 (PMC6158511; doi:10.1128/mSphere.00441-18)
Supplement: TABLE S5 [file sph005182646st5.pdf]

Database Level	AMR Class, Mechanism, or Group Value	Median normalized abundance in high *Bifidobacterium* samples (range)	Median normalized abundance in low *Bifidobacterium* samples (range)	Kruskal-Wallis test p-value
Class	Tetracyclines	0.0467 (0 – 0.815)	0.522 (0 – 2.51)	0.000709
Mechanism	Class A Betalactamases (Class Betalactamases)	0 (0 – 0.401)	0.242 (0 – 1.14)	0.000347
Mechanism	Tetracycline Resistance Ribosomal Proteins (Class Tetracyclines)	0.0123 (0 – 0.815)	0.444 (0 – 2.05)	0.000153
Group	TETQ (Class Tetracyclines)	0 (0 – 0.0455)	0.343 (0 – 2.05)	0.0000467

Supplemental Table S5
